# Blocking CIRP protects against acute pancreatitis by improving mitochondrial function and suppressing pyroptosis in acinar cells

**DOI:** 10.1038/s41420-024-01923-6

**Published:** 2024-03-27

**Authors:** Wuming Liu, Yifan Ren, Tao Wang, Mengzhou Wang, Yujia Xu, Jia Zhang, Jianbin Bi, Zheng Wu, Yuanyuan Zhang, Rongqian Wu

**Affiliations:** 1 National Local Joint Engineering Research Center for Precision Surgery and Regenerative Medicine, Shaanxi Provincial Center for Regenerative Medicine and Surgical Engineering, The First Affiliated Hospital of Xi’an Jiaotong University, Xi’an, China; 2 Department of Hepatobiliary Surgery, The First Affiliated Hospital of Xi’an Jiaotong University, Xi’an, China; 3 Department of General Surgery, The Second Affiliated Hospital of Xi’an Jiaotong University, Xi’an, China; 4 Department of Pathology, The First Affiliated Hospital of Wenzhou Medical University, Wenzhou, China; 5 Department of Gastroenterology, The Second Affiliated Hospital of Xi’an Jiaotong University, Xi’an, China; 6 Department of Oncology, The Second Affiliated Hospital of Xi’an Jiaotong University, Xi’an, China; 7 Department of Pediatrics, The First Affiliated Hospital of Xi’an Jiaotong University, Xi’an, China

**Keywords:** Acute inflammation, Stress signalling, Disease model

## Abstract

Acute pancreatitis (AP) continues to pose a major challenge as targeted therapeutic interventions are absent. Mitochondrial dysfunction and inflammasome-dependent pyroptosis are involved in the pathogenic mechanisms of AP. CIRP is a stress-response protein and a damage-associated molecular pattern (DAMP) molecule. In our previous studies, we discovered that excessive CIRP can directly damage pancreatic acinar cells. Nonetheless, the precise involvement of CIRP in AP is still unexplored. The primary aim of this study was to examine the potential involvement of CIRP in the development of pyroptosis and mitochondrial dysfunction in AP. To study this, an L-arginine-induced AP mouse model was used. Our results showed that Caspase-1-mediated pyroptosis and mitochondria-derived reactive oxygen species (ROS) were crucial factors in the occurrence of tissue damage and inflammation in AP. A substantial increase in the CIRP serum levels was observed in AP mice. Blocking CIRP by either CIRP gene knockout or systemic administration of C23, a competing inhibitor of CIRP, reduced ROS accumulation and pyroptosis in AP mice. These effects were associated with attenuated pancreatic injury and inflammation. In addition, CIRP-triggered mitochondrial dysfunction, autophagy impairment, and pyroptosis in pancreatic acinar cells were prevented by TAK242, an inhibitor of CIRP receptor TLR4. In conclusion, CIRP can induce mitochondrial dysfunction and pyroptosis in pancreatic acinar cells, and blocking CIRP may be a valuable approach to treating patients with AP.

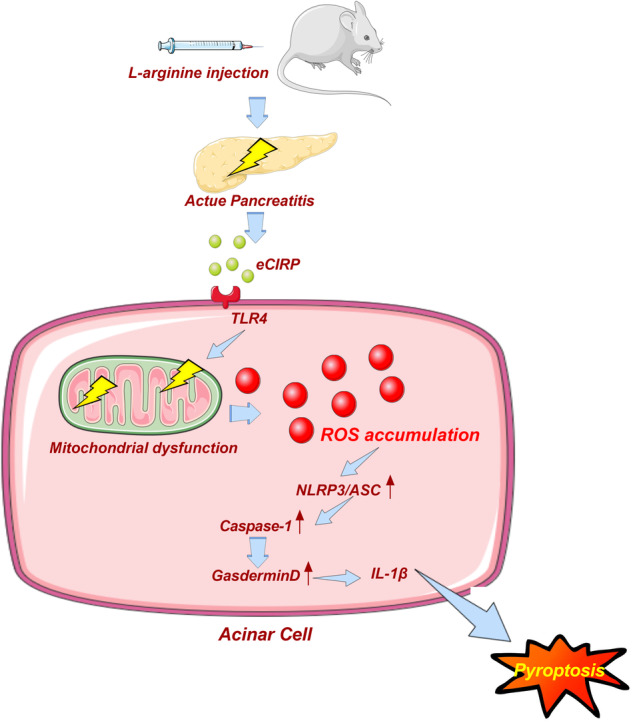

## Introduction

Acute pancreatitis (AP), a common inflammation disorder affecting the digestive system, is hallmarked by rapid development and high mortality [1]. AP is a prominent contributor to hospitalizations in the United States, accounting for an estimated 300,000 visits to emergency rooms annually [2]. The annual incidence of AP globally ranges between 30 and 40 cases per 100,000 individuals, resulting in significant financial burdens for those affected [3]. In particular, severe AP not only leads to necrosis of pancreatic tissue but also results in systemic inflammatory response syndrome (SIRS) and even multiple organ dysfunction syndrome (MODS) [4]. Severe AP is one of the main etiological factors for clinical pancreatitis-related mortality [1, 5]. Currently, effective targeted therapeutics are not available for treating pancreatitis in clinical settings, and AP is managed using supporting treatment.

Pyroptosis is an inflammation-triggered mode of programmed cell death [6]. In contrast to classical apoptosis and necrosis, pyroptosis is characterized by the formation of cell membrane pores, the swelling of the cytoplasm, the rupture of the cell membranes, the release of cytokines, and exacerbated systemic inflammatory responses [7]. Pyroptosis is Caspase-1-dependent and mediated mainly by the NLPR3 pathway. The recruitment of an apoptosis-related speck-like protein that binds to the cysteine protease Caspase-1 and the caspase recruitment domain (ASC) by inflammasome results in the formation of the NLRP3 inflammasome complex. This leads to the activation of GSDMD and interleukin (IL)-1β, which promotes several pathological processes [6]. Furthermore, pyroptosis is involved in the pathogenic mechanisms of several diseases, including some autoimmune diseases, tumors [8, 9], liver fibrosis [10], and atherosclerosis [11].

Cold-inducible RNA binding protein (CIRP) is an intracellular RNA chaperone protein produced from the cell when exposed to external stimuli, like low temperature or hypoxia [12]. Additionally, necrotic cells are capable of passively releasing CIRP [13]. Multiple studies have shown evidence that extracellular CIRP is involved in the pathogenesis of inflammatory disorders and functions as a damage-associated molecular pattern (DAMP) [12, 14]. Additionally, extracellular CIRP mediates inflammatory responses, such as hemorrhagic shock, sepsis [12], and trauma [15] through the TLR4/MD2 complex.

Mitochondria, which are the energy stations of cells, are the primary source of ATP production. Mitochondrial dysfunction can affect energy (ATP) metabolism, promote reactive oxygen species (ROS) production, and as a result, trigger oxidative stress [16, 17]. Oxidative stress can damage mitochondrial DNA and membrane structures and impair mitochondrial function [18]. Mitochondrial dysfunction is a pathological factor in AP [19]. Thus, AP can be mitigated by protecting mitochondrial function [19, 20]. ROS accumulation can also induce pyroptosis by activating NLRP3 [21, 22]. Mitochondrial dysfunction and impaired autophagy promote ROS accumulation [23]. Previously, we demonstrated that extracellular CIRP can directly damage pancreatic acinar cells [24]. Nonetheless, the precise role of CIRP in the pathogenesis of AP is unclear. This study hypothesized that CIRP mediates AP pathogenesis by promoting ROS accumulation, impairing mitochondrial function and autophagy, and upregulating pancreatic acinar cell pyroptosis.

Therefore, our primary focus is to study whether CIRP can cause excessive accumulation of ROS by damaging mitochondrial function and mitochondrial autophagy, thus activating the pyroptosis pathway in L-arginine-induced AP.

## Results

### Inhibition of pyroptosis alleviated pancreatic tissue damage in the L-arginine-induced AP model

**Fig. 1:**
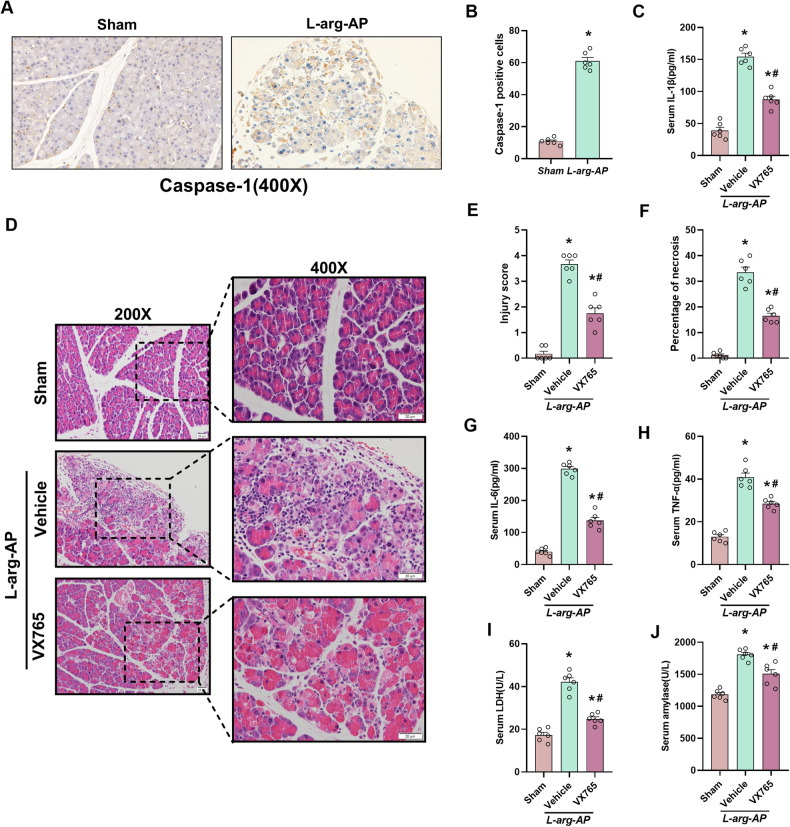
Pyroptosis suppression mitigated L-arginine-induced acute pancreatitis injury. L-Arginine-AP was induced by two hourly intraperitoneal injections of 4.0 g/kg L-arginine. A specific inhibitor for Caspase-1 (VX-765; 200 mg/kg) was intraperitoneally injected before the L-arginine injection and 1 h after the last L-arginine injection, respectively. The animals were sacrificed 72 h after the first injection of L-arginine. **A**, **B** Immunohistochemical analysis of the Caspase-1 expression in the pancreas. Scale bar: 20 µm. **C** Serum interleukin (IL)-1β levels. **D** Representative images of pancreatic sections subjected to hematoxylin and eosin (HE) staining. Scale bar: 20 µm. **E** Pancreatic injury scores. **F** Percentages of necrotic areas. **G** Serum interleukin (IL)-6 levels. **H** Serum tumor necrosis factor (TNF)-α levels. **I** Serum lactate dehydrogenase (LDH) levels. **J** Serum amylase levels. *n* = 6/group. *p < 0.05 versus sham group; #*p* < 0.05 versus vehicle group. Data are expressed as means ± SEM.

An L-arginine-induced AP mouse model was established to examine the involvement of CIRP and pyroptosis in the pathogenesis of AP. Previous studies have reported that pancreatic tissue damage peaked at 72 h post-L-arginine intraperitoneal injection [20]. Immunohistochemical (IHC) analysis revealed that the expression of Caspase-1, a pyroptosis marker protein, was remarkably (*p* < 0.05) upregulated in AP mice (Fig. 1A, B). Additionally, the serum concentration of IL-1β, an inflammatory effector of the pyroptosis pathway, was remarkably upregulated in AP mice (Fig. 1C). These findings indicate that the occurrence of pancreatitis was accompanied by pyroptosis induction. Mice were intraperitoneally administered with VX-765 (200 mg/kg body weight), a Caspase-1 selective inhibitor, which is a key pyroptosis-related protein. In AP animals, the extent of necrosis, infiltration of inflammatory cells, and pancreatic tissue edema was increased relative to that in the sham group, as shown by HE staining (Fig. 1D). Nevertheless, VX765 administration mitigated pancreatic tissue damage and markedly decreased injury scores and necrosis area in AP mice (Fig. 1E, F). Similarly, VX765 administration mitigated the release of necrosis factor LDH and pro-inflammatory markers TNF-α and IL-6 in mice serum (Fig. 1G–I). Furthermore, the VX765 treatment alleviated the elevated serum pancreatic amylase level (Fig. 1J).

### Inhibition of mitochondrion-derived ROS suppressed pancreatic pyroptosis and tissue damage in the AP model

**Fig. 2:**
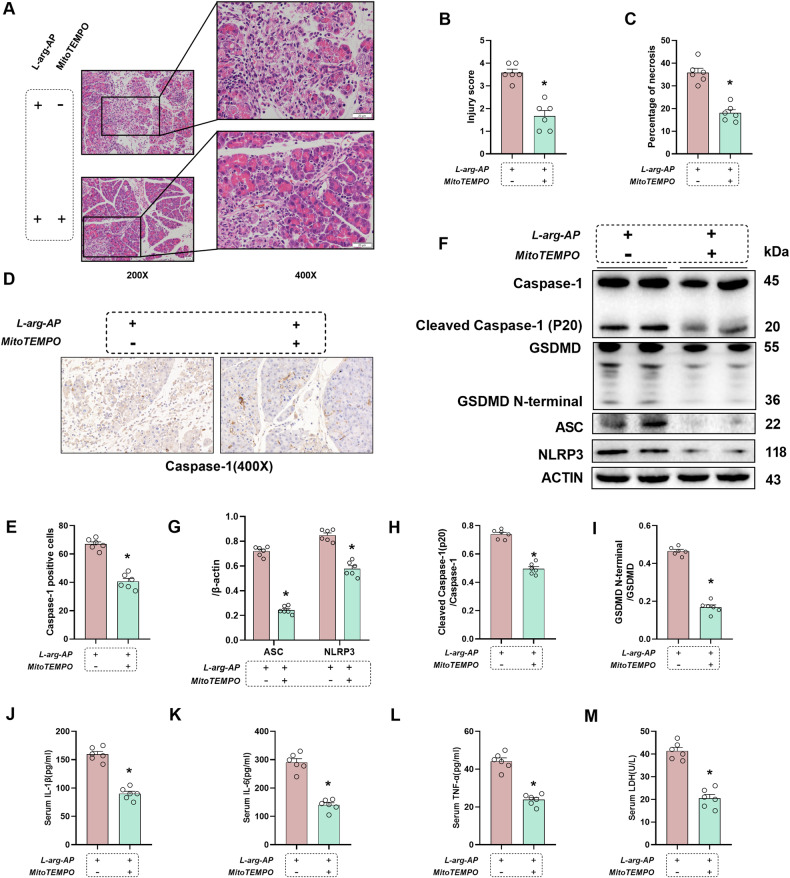
Pancreatic tissue injury and pyroptosis were alleviated by ROS inhibition in L-arginine-induced acute pancreatitis. L-Arginine-AP was induced by two hourly intraperitoneal injections of 4.0 g/kg L-arginine. A specific scavenger of mitochondrial superoxide (MitoTEMPO,20 mg/kg, Sigma) was intraperitoneally injected 1 h before L-arginine injection. The animals were sacrificed 72 h after the first injection of L-arginine. **A** Representative images of pancreatic sections subjected to hematoxylin and eosin (HE) staining. Scale bar: 20 µm. **B** Pancreatic injury scores. **C** Percentages of necrotic areas. **D**, **E** Immunohistochemical analysis of the Caspase-1 expression in the pancreas. Scale bar: 20 µm. **F**−**I** Western blot analysis of Caspase-1, ASC, NLRP3, and GSDMD in the pancreas. **J** Serum interleukin (IL)-1β levels. **K** Serum interleukin (IL)-6 levels. **L** Serum tumor necrosis factor (TNF)-α levels. **M** Serum lactate dehydrogenase (LDH) levels. *n* = 6/group. *p < 0.05 versus L-arginine-AP with MitoTEMPO-untreated group. Data are expressed as means ± SEM.

To further investigate the correlation between ROS and pyroptosis in AP, mice were administered with a specific scavenger of mitochondrial superoxide (MitoTEMPO; 20 mg/kg body weight; Sigma) 1 h before L-arginine injection. HE staining revealed that the pancreatic damage and necrotic area in the MitoTEMPO-treated group were considerably ameliorated in comparison to those in the vehicle group (Fig. 2A–C). The results of serum LDH analysis validated these findings (Fig. 2M). IHC analysis revealed that the Caspase-1 level was substantially upregulated in AP mice but was downregulated upon MitoTEMPO treatment (Fig. 2D, E). Western blotting analysis revealed that MitoTEMPO treatment markedly downregulated Caspase-1 expression and other pyroptosis activation-associated proteins, NLRP3, ASC, and GSDMD in AP mice (Fig. 2F–I). Additionally, MitoTEMPO mitigated the upregulated serum levels of IL-1β and systemic pro-inflammatory indicators (TNF-α and IL-6), suggesting that ROS suppression alleviated pyroptosis in the L-arginine-induced AP mouse model (Fig. 2J–L).

Pancreatic AR42J cells (5 × 10^5^ cells/well) were pretreated with the ROS scavenger n-acetylcysteine (NAC, Sigma-Aldrich, 5 nM) for 2 h and treated with cerulein (10 nmol/L) + LPS (10 µg/mL) for 24 h to verify the correlation between ROS and pyroptosis in vitro. Western blotting analysis revealed that the Caspase-1, ASC, NLRP3, and GSDMD expression were remarkably upregulated in the cerulein + LPS-treated group, suggesting that pyroptosis was activated in the in vitro AP model. However, these proteins were shown to be downregulated considerably with the intervention of NAC, implying that suppressing ROS can reduce the pyroptosis level of pancreatic acinar cells (Supplementary Fig. 2A–D). ROS are crucial in triggering pyroptosis during the pathogenesis of AP, as validated by our findings.

### CIRP deficiency attenuated pancreatic pyroptosis and tissue injury in the L-arginine-induced AP mouse model

**Fig. 3:**
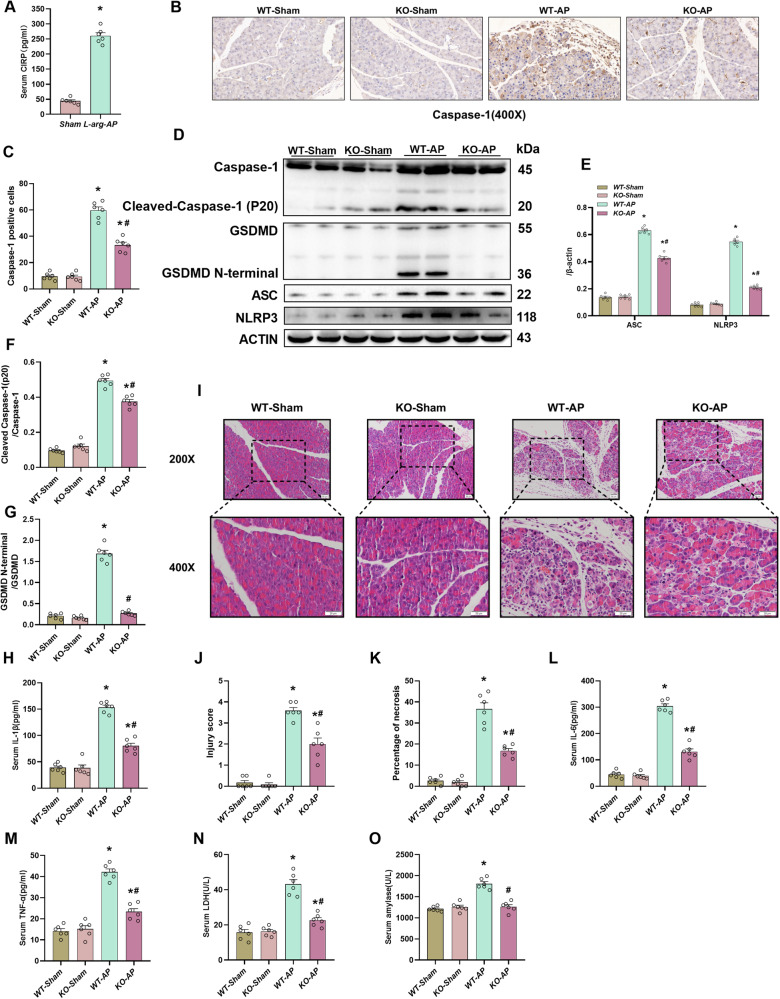
CIRP KO attenuated pancreatic pyroptosis and tissue damage in the L-arginine-induced AP model. L-Arginine-AP was induced by two hourly intraperitoneal injections of 4.0 g/kg L-arginine. The animals were sacrificed 72 h after the first injection of L-arginine. **A** Serum CIRP levels. **B**, **C** Immunohistochemical analysis of pancreatic Caspase-1 expression in wild-type and *CIRP*^*−/−*^ mice. Scale bar: 20 µm. **D**−**G** Western blotting analysis of Caspase-1, ASC, NLRP3, and GSDMD in the pancreas. **H** Serum interleukin (IL)-1β levels. **I** Representative images of pancreatic tissues subjected to hematoxylin and eosin (HE) staining. Scale bar: 20 µm. **J** Pancreatic injury scores. **K** Percentages of necrotic areas. **L** Serum interleukin (IL)-6 levels. **M** Serum tumor necrosis factor (TNF)-α levels. **N** Serum lactate dehydrogenase (LDH) levels. **O** Serum amylase levels. *n* = 6/group. *p < 0.05 versus sham group; #*p* < 0.05 versus WT-AP group. Data are expressed as means ± SEM.

The serum CIRP levels in a mouse model of AP triggered by L-arginine were measured to investigate the function of CIRP in AP. The serum CIRP level was remarkably upregulated in AP mice (Fig. 3A). Subsequently, pancreatic tissues were subjected to Caspase-1 immunohistochemical analysis to additionally examine the involvement of CIRP in pyroptosis in AP. The pancreatic levels of Caspase-1 were significantly upregulated in AP mice. However, the upregulation of Caspase-1 was significantly suppressed in *CIRP*^*−/−*^ mice (Fig. 3B, C). Similarly, the expression levels of other pyroptosis activation-associated proteins ASC, GSDMD, and NLRP3 were significantly higher in the acute pancreatitis model. Interestingly, the above phenomena were reduced in CIRP^-/-^ mice This suggests that CIRP promotes pyroptosis in the L-arginine-induced AP model (Fig. 3D–G). NLRP3 can also recruit Caspase-1 through ASC. Activated Caspase-1 enhances the maturation of IL-1β by cleaving pro-IL-1β. The IL-1β levels were remarkably elevated in AP mice, and this upregulation was mitigated in *CIRP*^*−/−*^ mice (Fig. 3H). HE staining revealed that *CIRP* KO significantly alleviated pancreatic tissue damage in AP mice (Fig. 3I–K), as well as suppressed the upregulated serum levels of LDH, pro-inflammatory indicators (TNF-α and IL-6) (Fig. 3L–N), and amylase (Fig. 3O). Please note that serum amylase levels in CIRP KO mice induced by L-Arginine were consistent with the control mice, but HE staining still showed significant damage in the pancreatic tissue of these animals. These findings are consistent with pathophysiological changes of L-arginine-induced AP models. Following L-arginine injection, amylase concentrations in the serum peaked between 12 and 24 h but recovered to normal between 24 and 48 h later. After 72 h, however, the degree and severity of necrotic alterations in the exocrine tissue of the pancreas accompanied by infiltration of inflammatory cells peaked [25].

### CIRP deficiency improved mitochondrial function and suppressed ROS accumulation in the L-arginine-induced AP mouse model

**Fig. 4:**
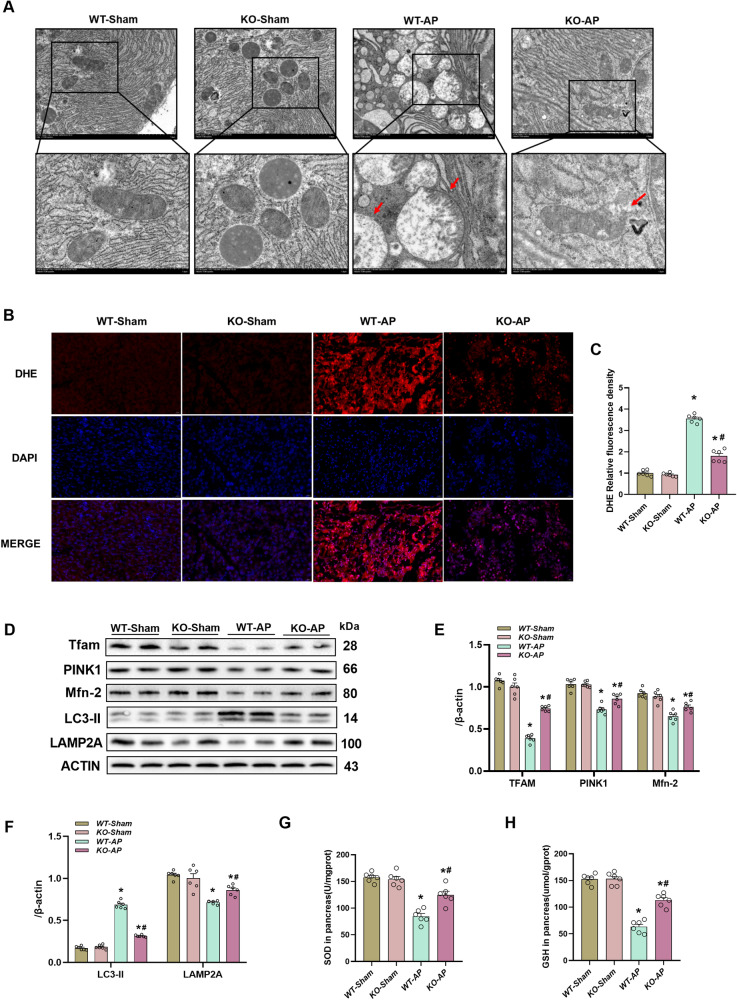
CIRP knockout improved mitochondrial function and reduced ROS accumulation in the L-arginine-induced AP model. **A** Ultrastructural alterations in the pancreas (Transmission electron microscopy). Scale bars:2 µm and 5 µm. **B** Representative images of pancreatic tissues subjected to dihydroethidium (DHE) immunofluorescence staining. Scale bar: 20 µm. **C** DHE relative fluorescence density analysis. **D**–**F** Western blotting analysis of Tfam, PINK1, Mfn2, LAMP2A, and LC3-II in the pancreas; (**G**) Superoxide dismutase (SOD) levels in the pancreatic tissue. **H** Glutathione (GSH) levels in the pancreatic tissue. *n* = 6/group. *p < 0.05 versus sham group; #*p* < 0.05 versus WT-AP group. Data are expressed as means ± SEM.

The ROS levels were examined in AP mice to ascertain the potential ROS function in pyroptosis in AP. DHE staining revealed that the pancreatic ROS levels were substantially upregulated in AP mice, and this upregulation was mitigated in *CIRP*^*−/−*^ mice (Fig. 4B, C). Consistently, the pancreatic levels of SOD and GSH were markedly downregulated in AP mice, and this downregulation was mitigated in *CIRP*^*−/−*^ mice (Fig. 4G, H). Mitochondrial dysfunction has been observed to be linked to excessive oxidative stress triggered by ROS [16]. Thus, maintaining physiological mitochondrial function requires healthy mitochondrial dynamics, which include fission, biogenesis, and fusion. Notably, the levels of Tfam and PINK1 (two critical regulators of mitochondrial biogenesis), as well as those of Mfn2 (a regulator of mitochondrial fusion and mitophagy), were significantly downregulated in AP mice, as evidenced by Western blotting analysis. Transmission electron microscopy (TEM) revealed that following the induction of AP in mice, there was an increase in the swelling of mitochondria, as well as condensation, disruption, or cristae loss (Fig. 4A). These L-arginine-induced changes were suppressed in *CIRP*^*−/−*^ mice (Fig. 4D, E). Furthermore, mitochondrial dysfunction can be alleviated by suppressing the accumulation of ROS via mitophagy [26]. The LAMP2A (autophagy function-related protein) expression was remarkably downregulated in AP mice, whereas that of LC3-II was significantly upregulated, indicating that autophagy function was impaired in AP mice. However, these changes were mitigated in *CIRP*^*−/−*^ mice, suggesting that the *CIRP* KO alleviated mitochondrial autophagy dysfunction (Fig. 4D–F).

### C23 administration suppressed pancreatic pyroptosis and tissue damage in the L-arginine-induced AP mouse model

**Fig. 5:**
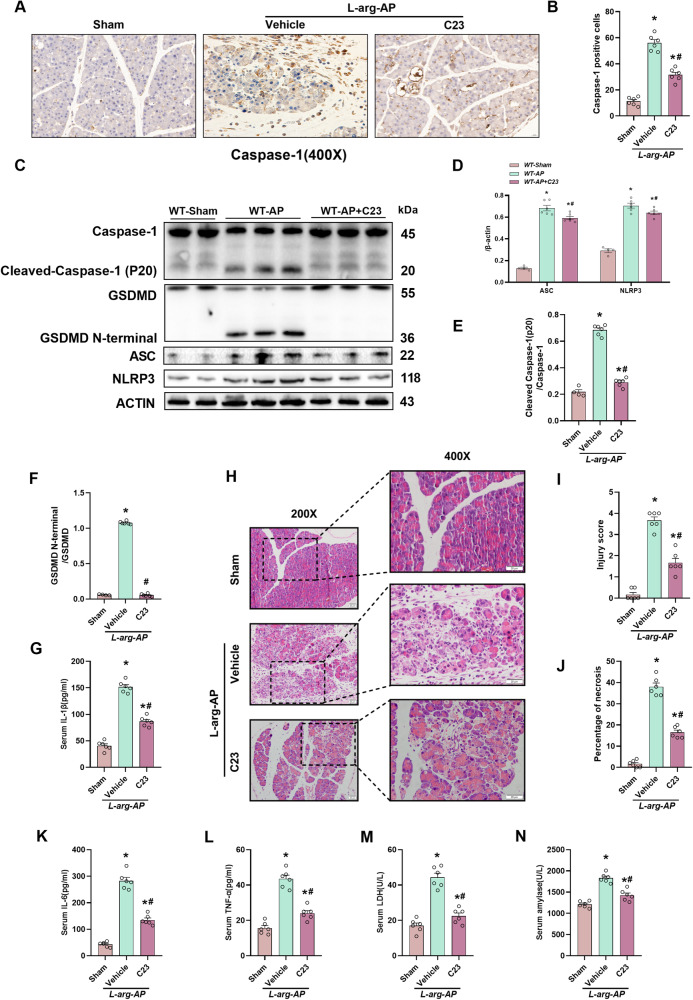
C23 administration attenuated pancreatic pyroptosis and tissue damage in the L-arginine-induced AP model. L-Arginine-AP was induced by two hourly intraperitoneal injections of 4.0 g/kg L-arginine. The animals were sacrificed 72 h after the first injection of L-arginine. C23 (8 mg/kg) was administered 2 h after the last L-arginine injection was administered by intraperitoneal injection. Mice in the sham group were intraperitoneally administered with the same volume of physiological saline. **A**, **B** Immunohistochemical analysis of the Caspase-1 expression in the pancreas. Scale bar: 20 µm. **C**−**F** Western blotting analysis of Caspase-1, ASC, NLRP3, and GSDMD in the pancreas. **G** ©(IL)-1β levels. **H** Representative images of pancreatic sections subjected to hematoxylin and eosin (HE) staining. Scale bar: 20 µm. **I** Pancreatic injury scores. **J** Percentages of necrotic areas. **K** Serum interleukin (IL)-6 levels. **L** Serum tumor necrosis factor (TNF)-α levels. **M** Serum lactate dehydrogenase (LDH) levels. **N** Serum amylase levels. *n* = 6/group. *p < 0.05 versus sham group; #*p* < 0.05 versus vehicle group. Data are expressed as means ± SEM.

Animals were treated with C23, a competing inhibitor of CIRP that prevents the binding of CIRP to its receptor TLR4, to further ascertain the involvement of CIRP in AP-related pyroptosis. IHC analysis revealed that C23 significantly downregulated the pancreatic expression levels of Caspase-1 (Fig. 5A, B). Additionally, C23 mitigated the L-arginine-induced upregulation of Caspase-1, NLRP3, ASC, and GSDMD (Fig. 5C–F). Furthermore, C23 suppressed the L-arginine-induced upregulation of serum IL-1β (Fig. 5G), LDH, IL-6, TNF-α, and pancreatic amylase and pancreatic damage (Fig. 5H–N).

### C23 administration improved mitochondrial function and suppressed ROS accumulation in the L-arginine-induced AP mouse model

**Fig. 6:**
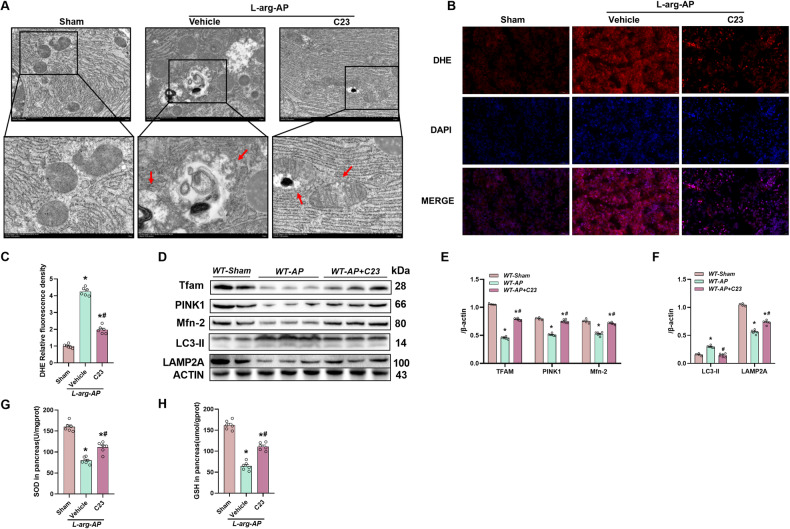
C23 administration improved mitochondrial function and reduced ROS accumulation in the L-arginine-induced AP model. **A** Ultrastructural alterations in the pancreas (Transmission electron microscopy). Scale bars:2 µm and 5 µm. **B** Representative images of pancreatic sections subjected to dihydroethidium (DHE) immunofluorescence analysis. Scale bar: 20 µm. **C** DHE relative fluorescence intensity analysis. **D**−**F** Western blotting analysis of Tfam, PINK1, Mfn2, LAMP2A, LC3-II in the pancreas; (**G**) Superoxide dismutase (SOD) levels in the pancreatic tissue. **H** Glutathione (GSH) levels in the pancreatic tissue. *n* = 6/group. *p < 0.05 versus sham group; #*p* < 0.05 versus vehicle group. Data are expressed as means ± SEM.

DHE staining revealed that C23 mitigated the L-arginine-induced upregulation of pancreatic ROS levels (Fig. 6B, C) and downregulation of pancreatic SOD and GSH levels, suggesting that the levels of antioxidants in pancreatic tissue were restored by C23 (Fig. 6G, H). TEM also revealed that mitochondrial swelling and condensation, disruption, or loss of cristae were mitigated by C23 administration (Fig. 6A). Western blotting analysis revealed that C23 suppressed the L-arginine-triggered downregulation of Tfam, PINK1, and Mfn2 (Fig. 6D, E), upregulation of LAMP2A and LC3-II, and impairment of mitochondrial autophagy function (Fig. 6D, F).

### C23 mitigated cerulein + LPS-induced mitochondrial dysfunction, autophagy impairment, ROS accumulation, and pyroptosis in cultured pancreatic acinar cells

**Fig. 7:**
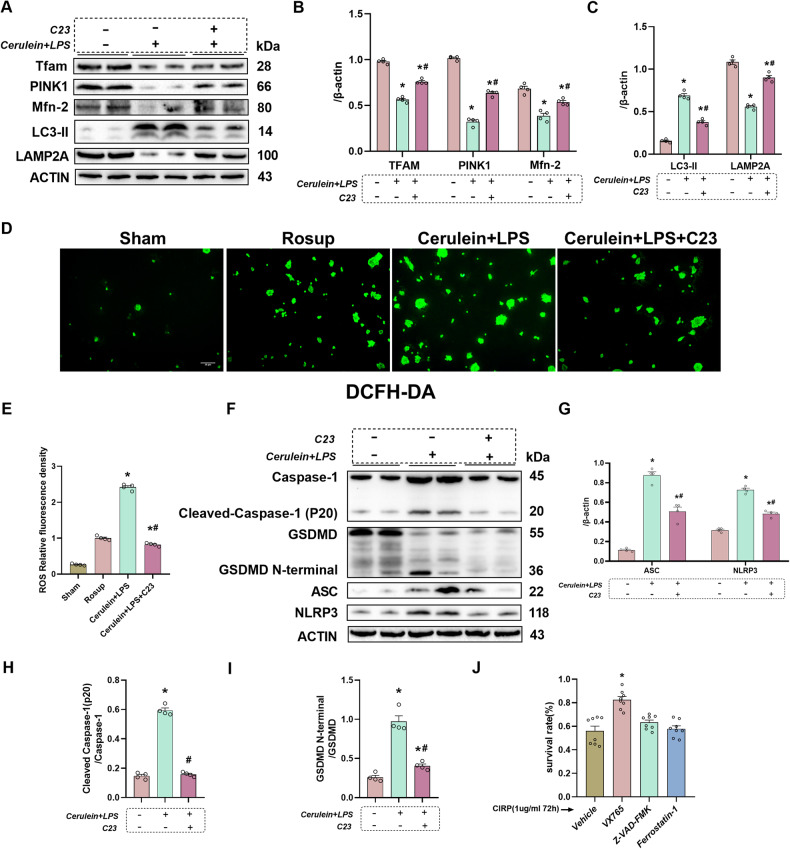
C23 attenuated cerulein + LPS-induced mitochondrial dysfunction, autophagy impairment, ROS accumulation and pyroptosis in cultured pancreatic acinar cells. Pancreatic AR42J cells (5 × 10^5^ cells/well) were treated with cerulein (10 nmol/L) + LPS (10 µg/mL) for 24 h. Pancreatic AR42J cells (5 × 10^5^ cells/well) in the C23-treated group were pretreated with C23 (450 ng/mL) (GRGFSRGGGDRGYGG synthesized from GenScript, Piscataway, NJ; dissolved in phosphate-buffered saline) for 1 h. **A**–**C** Western blotting analysis of Tfam, PINK1, Mfn2, LAMP2A, LC3-II in pancreatic AR42J cells treated with cerulein + LPS for 24 h after pretreatment with or without C23. **D** Representative images of ROS immunofluorescence analysis in pancreatic AR42J cells. Scale bar: 50 µm. **E** ROS relative fluorescence intensity analysis. **F**−**I** Western blotting analysis of Caspase-1, ASC, NLRP3, and GSDMD in pancreatic AR42J cells treated with cerulein + LPS for 24 h after pretreatment with or without C23. **J** The survival rates of AR42J cells were evaluated using the 3-(4,5-dimethylthiazol-2-yl)-2,5-diphenyl tetrazolium bromide (MTT) assay. *n* = 4/group. *p < 0.05 versus sham group; #*p* < 0.05 versus Cerulein+LPS group. Data are expressed as means ± SEM.

Next, in vitro experiments were performed with pancreatic AR42J cells. Subsequently, pancreatic AR42J cells (5 × 10^5^ cells/well) were subjected to cerulein (10 nmol/L) + LPS (10 µg/mL) for 24 h and used to simulate AP in vitro. Cerulein is a cholecystokinin (CCK) analog, which provokes digestive enzyme secretion. Both cerulein alone and cerulein + LPS are widely used methods to induce pancreatitis [25]. Cerulein alone usually induces mild acute pancreatitis, while cerulein + LPS can induce severe acute pancreatitis under both in vivo and in vitro conditions. Here, we found that the CIRP level in the supernatant of AR42J cells was significantly increased after cerulein + LPS treatment, which was in agreement with the findings of investigations conducted in vivo (Supplementary Fig 1). Additionally, the Tfam, PINK1, and Mfn2 expressional levels were remarkably downregulated in the cerulein + LPS-treated group (Fig. 7A, B). Meanwhile, the expression levels of autophagy function-related protein LAMP2A were significantly downregulated, whereas those of LC3-II were considerably upregulated in the cerulein + LPS-treated group (Fig. 7A, C). Treatment with C23 alleviated cerulein + LPS-induced mitochondrial dysfunction and autophagy impairment in acinar cells (Fig. 7A–C). The ROS levels in pancreatic AR42J cells were examined using the ROS assay kit. Treatment with C23 significantly mitigated the cerulein + LPS-induced upregulation of ROS accumulation in AR42J cells (Fig. 7D, E).

Moreover, the level of pyroptosis-related proteins Caspase-1, ACS, NLRP3, and GSDMD remarkably increased in vitro and alleviated by using CIRP’s competing inhibitor C23 (Fig. 7F–I), which agrees with the in vivo finding. Next, pancreatic AR42J cells were pretreated with different cell death inhibitors and stimulated with recombinant CIRP. The MTT assay illustrated that treatment with the pyroptosis inhibitor VX765 significantly increased cell survival rates (Fig. 7J). Based on these findings, it is evident that CIRP mediates pyroptosis in the in vitro AP model.

### CIRP directly induced mitochondrial dysfunction, autophagy impairment, ROS accumulation, and pyroptosis in cultured pancreatic acinar cells

**Fig. 8:**
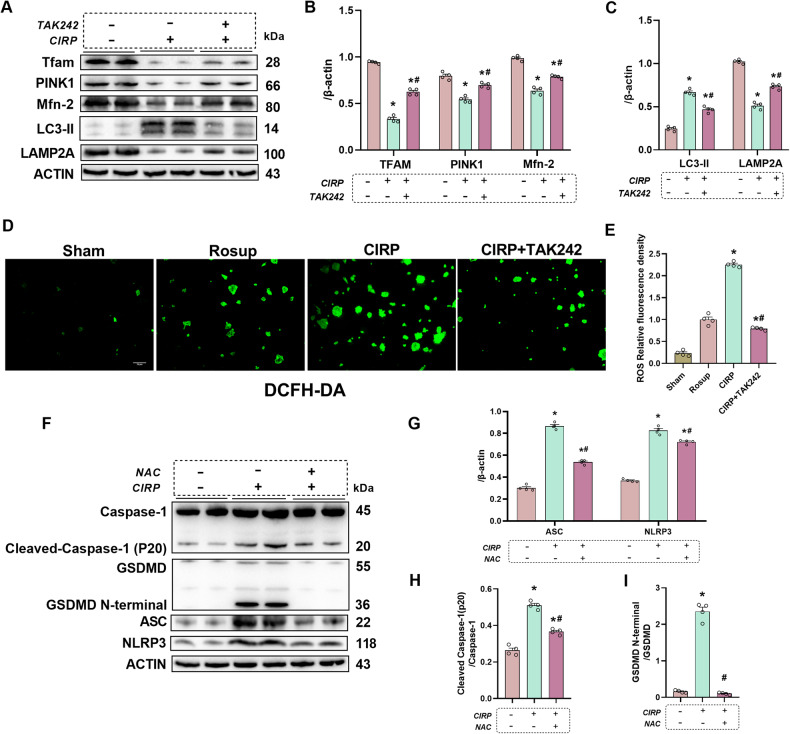
CIRP directly induced mitochondrial dysfunction, autophagy impairment, ROS accumulation and pyroptosis in cultured pancreatic acinar cells. Pancreatic AR42J cells (5 × 10^5^ cells/well) were treated with recombinant CIRP (1.5 µg/mL) for 24 h. Pancreatic AR42J cells in the NAC-treated group were pretreated with the ROS scavenger n-acetylcysteine (NAC; 5 nM) (BP907, Sigma, USA) for 2 h. Pancreatic AR42J cells in the TAK242-treated group were pretreated with TAK-242 (500 µM) for 24 h. **A**–**C** Western blotting analysis of Tfam, PINK1, Mfn2, LAMP2A, LC3-II in pancreatic AR42J cells treated with recombinant CIRP for 24 h after pretreatment with or without TAK242. **D** Representative images of ROS immunofluorescence analysis in pancreatic AR42J cells. Scale bar: 50 µm. **E** ROS relative fluorescence intensity analysis. **F**−**I** Western blotting analysis of Caspase-1, ASC, NLRP3, and GSDMD in pancreatic AR42J cells treated with recombinant CIRP for 24 h after pretreatment with or without NAC. *n* = 4/group. *p < 0.05 versus sham group; #*p* < 0.05 versus CIRP group. Data are expressed as means ± SEM.

TAK242 was administered to the AR42J cells to better explore the specific mechanisms that are involved in CIRP. In particular, 1.5 µg/mL recombinant CIRP was administered to AR42J cells (5 × 10^5^ cells/well) for 72 h after pretreatment with or without TAK242 (500 µM) for 24 h. Notably, TAK242 upregulated the Tfam, Mfn2, and PINK1 expression levels in AR42J cells, as shown by Western blotting analysis results (Fig. 8A, B). Additionally, TAK242 alleviated autophagy impairment by upregulating the LAMP2A levels and downregulating the LC3-II levels (Fig. 8A, C). Furthermore, TAK242 significantly downregulated ROS accumulation in AR42J cells (Fig. 8D, E). These results suggest that CIRP promotes ROS accumulation, mitochondrial dysfunction, and autophagy injury in the in vitro AP model through its receptor, TLR4.

Pancreatic AR42J cells (5 × 10^5^ cells/well) were pretreated with NAC (Sigma-Aldrich; 5 nM) for 2 h followed by subsequent treatment with 1.5 µg/mL recombinant CIRP for 24 h to verify the correlation between ROS and pyroptosis in vitro. Accordingly, the expression levels of related proteins Caspase-1, ASC, NLRP3, and GSDMD were substantially upregulated in pancreatic AR42J cells treated with recombinant CIRP as shown by Western blotting, suggesting that CIRP directly activated pyroptosis in pancreatic acinar cells. Treatment with NAC significantly mitigated the recombinant CIRP-induced upregulation of Caspase-1, ASC, NLRP3, and GSDMD, indicating that the inhibition of ROS can suppress pyroptosis in pancreatic AR42J cells (Fig. 8F–I). These findings indicate that ROS promotes pyroptosis in AP.

## Discussion

This study demonstrated that pyroptosis is activated in AP. Inhibition of pyroptosis can significantly reduce pancreatic tissue injury, the injury score and necrotic area, and the release of injury-related factor LDH and other pro-inflammatory factors to alleviate the progression of AP. Inhibition of CIRP in various ways alleviates pyroptosis in both in vitro and in vivo AP models by relieving mitochondrial dysfunction, mitochondrial autophagy damage, and reactive oxygen species accumulation. CIRP can not only aggravate tissue injury by damaging the mitochondrial function but also hinder the clearance of ROS by damaging the autophagy function of mitochondria so that excessive ROS can further activate NLRP3 to mediate the pyroptosis in AP. These results reveal a novel CIRP-mediated acute pancreatitis injury pathway and provide a new target for future treatment.

AP, an inflammatory disease without specific treatment, is characterized by pancreatic tissue damage and necrosis as well as the secretion of several inflammatory cytokines into the bloodstream, leading to a cascade reaction, which, in extreme circumstances, may lead to multiple organ failure and systemic inflammatory response syndrome [27, 28]. Existing research has shown that serum CIRP levels are substantially upregulated after the onset of AP and exhibit a favorable correlation with the severity of AP [29]. This was confirmed in this study using the L-arginine-induced AP murine model. According to Linders J et al., the extracellular CIRP is an essential factor in the formation of neutrophil extracellular traps in AP [30]. Xu Q et al. demonstrated that emodin alleviates lung injury in AP by blocking the CIRP-mediated NLRP3 pathway [31]. CIRP, an intracellular RNA chaperone protein, is transferred from the intracellular environment to the extracellular environment when exposed to external stimuli, such as hypoxia and low temperature [12]. CIRP can also be passively released extracellularly from necrotic cells [13]. Extracellular CIRP functions as a DAMP and regulates the pathogenesis of various inflammatory diseases through TLR4. Previous studies have reported that TLR4 expression is significantly upregulated in acinar cells during AP [32]. Thus, CIRP may mediate damage by binding to TLR4 expressed on acinar cells. C23, an oligopeptide derived from CIRP, competitively inhibits CIRP binding to TLR4 [33, 34]. In this study, *CIRP* KO and C23 were used to block CIRP to elucidate its action mechanisms. The findings of this study demonstrated that CIRP inhibition significantly alleviates AP.

Mitochondrial damage is a critical pathogenic process in AP [19]. Recently, Biczo et al. reported that mitochondrial dysfunction mediates hyperamylase, trypsinogen activation, necrosis, vacuolization, and inflammation in L-arginine-mediated AP [19]. CIRP is also recognized for its involvement in promoting mitochondrial dysfunction. Zheng X et al. demonstrated that CIRP mediates mitochondrial dynamics disorder, exacerbates intracellular oxidative stress, upregulates NADPH oxidase *via* the TLR-4/MyD88 pathway, and consequently promotes apoptotic activity in HK-2 cells [35]. Li Z et al. demonstrated that cold-inducible RNA-binding proteins induce mitochondrial DNA breakage through the TLR4 signaling pathway and regulate macrophage death after trauma [15]. Consistent with the results of our previous studies, the expression of the mitochondrial function-associated proteins Tfam and Mfn2 was significantly downregulated in the L-arginine-induced AP mouse model [36], indicating that mitochondrial function was significantly impaired in AP. The L-arginine-induced effects were significantly mitigated in *CIRP*^*−/−*^ and C23-treated mice. Similar findings were obtained in the in vitro AP model. Treatment with recombinant CIRP significantly downregulated the expression of mitochondrial function-related proteins in acinar cells. TAK242, a specific inhibitor of TLR4, significantly suppressed the effect of recombinant CIRP, suggesting that CIRP promotes mitochondrial dysfunction in pancreatic acinar cells through TLR4. Furthermore, ROS synthesis primarily occurs in mitochondria. The dysfunction of mitochondria promotes ROS production, exacerbating mitochondrial dysfunction [37].

Mitochondrial autophagy is a cellular response that is both conserved and adaptable and is responsible for the selective elimination of mitochondria that are defective or damaged through the autophagosome [38]. Autophagy in mitochondria is essential for the removal of ROS since mitochondria are the principal generator of ROS [39]. Damaged mitochondria can lead to impaired ROS clearance [26]. Liu Z et al. demonstrated that the overexpression of mitochondrial ROS promotes pyroptosis in hepatocytes by impairing mitochondrial autophagy [40]. AP is often accompanied by impaired autophagy [41, 42]. A previous study reported that the dysregulation of the autophagic pathway in AP contributes to the downregulation of LAMP2A, a marker of enzyme-body formation [43]. The lack of lysosomal involvement inhibits the autophagy pathway and the clearance of autophagosomes, resulting in the upregulation of LC3-II [44]. In this study, the expression of Lamp2a was significantly downregulated, whereas that of LC3-II was significantly upregulated in the in vivo and in vitro AP models, suggesting that the autophagy function of mitochondria was impaired. However, autophagy was significantly downregulated in *CIRP* KO and C23-treated mice. Thus, it is evident from these findings that CIRP promotes autophagy injury in AP. Additionally, treatment with TAK242 mitigates recombinant CIRP-induced autophagy injury in pancreatic acinar cells, suggesting that CIRP promotes autophagy injury through TLR4. As demonstrated in existing literature, the PINK1 expression is upregulated after autophagy activation [45]. Based on these findings, it seems that the PINK1 and Mfn2 expression levels are particularly significant for the activation of mitophagy [46, 47]. In this study, the PINK1 and Mfn2 expression levels were remarkably downregulated in the in vivo and in vitro AP models.

CIRP is reported to promote ROS accumulation. Sakurai T et al. demonstrated that the lack of CIRP downregulates ROS accumulation and consequently suppresses the onset of liver cancer [48]. Additionally, Liu W et al. found that rhCIRP up-regulated the expression of gp91phox and P47phox in HL-7702 cells, caused apoptosis, elevated ROS levels, and stimulated the secretion of inflammatory factors [49]. Immunofluorescence analysis revealed that the ROS levels were significantly upregulated in the L-arginine-induced AP mouse model, while the antioxidant indices (SOD and GSH) were significantly downregulated, suggesting that AP was accompanied by oxidative stress induction. *CIRP* KO or C23 treatment significantly mitigated the effects of L-arginine in mice. C23 treatment suppressed ROS accumulation in the in vitro AP model. TAK242 suppressed recombinant CIRP-induced ROS accumulation in pancreatic acinar cells. Based on these findings, CIRP promotes ROS accumulation in AP through Tlr4. The formation of ROS is a typical upstream process that is involved in the activation of the NLRP3 inflammasome. Previous studies have reported that ROS promotes pyroptosis. Wu X et al. demonstrated that nicotine is responsible for the promotion of atherosclerosis via the ROS-NLRP3-elicited pyroptosis of endothelial cells [21]. Liu Z et al. reported that the accumulation of mitochondrial ROS resulting from impaired mitophagy enhances pyroptosis in hepatocytes [40]. In this study, the marker proteins of the pyroptosis pathway Caspase-1, NLRP3, and GSDMD, the marker proteins of the pyroptosis pathway, were significantly downregulated in the in vitro and in vivo AP models upon treatment with a ROS scavenger. Additionally, the ROS scavenger significantly mitigated recombinant CIRP-induced pyroptosis in AR42J cells. These findings indicate that ROS can activate the Caspase1-dependent pyroptosis pathway through NLRP3 in AP. The excessive accumulation of ROS may be caused by the impaired autophagy function of mitochondria is mediated by CIRP. Thus, CIRP released to the extracellular environment in AP serves as a DAMP to mediate mitochondrial dysfunction in pancreatic acinar cells, exacerbating tissue damage and promoting ROS accumulation.

Pyroptosis, a novel mode of programmed cell death that is distinct from traditional cell necrosis and apoptosis, is distinguished by the development of cell membrane pores and the secretion of the effector cytokine IL-1β [7]. Caspase-1, which has been activated, is involved in cleaving the GSDMD protein molecule and initiating the oligomerization of the amino terminus (GSDMD-N), thereby promoting the formation of plasma membrane pores, the secretion of IL-1β and IL-18, and consequently inducing pyroptosis, which is implicated in the pathogenesis of systemic inflammation [50]. It has been illustrated in a series of studies that pyroptosis which is reliant on the NLRP3 inflammasome contributes to the pathophysiology of AP. Gao L et al. demonstrated that AP involves NLRP3 inflammasome and GSDMD activation-mediated pyroptosis and systemic inflammation [51]. Wang J et al. reported that cathepsin B exacerbates the severity of AP through the activation of NLRP3 inflammasome and enhancement of Caspase-1-elicited pyroptosis [52]. Herein, the expression of Caspase-1, a pyroptosis-related marker protein, was significantly upregulated in the L-arginine-induced AP mouse model. Treatment with pyroptosis inhibitors alleviated AP-related tissue damage, indicating that pyroptosis is involved in the pathogenic mechanisms of AP. However, *CIRP* KO and C23 treatment significantly alleviated pyroptosis in AP. Similarly, C23 treatment suppressed pyroptosis in the in vitro AP models. As demonstrated by the MTT assay, the pyroptosis inhibitor VX765 significantly increased the survival rate of recombinant CIRP-treated pancreatic acinar cells. These results suggest that CIRP induces acinar cell damage by promoting pyroptosis, suggesting that CIRP suppression alleviates pyroptosis in AP.

Our research has a few drawbacks. First, the CIRP-deficient mice employed in this study are global knockout. Although we proved the key role of CIRP in the pathogenic mechanism of AP by using these mice, the source of extracellular CIRP could not be identified. A recent review article suggested that both activated macrophages and necrotic cells can be the sources of extracellular CIRP [13]. In the future, macrophage-specific CIRP knockout mice and pancreatic acinar cell-specific CIRP knockout mice should be used to pinpoint the major source of CIRP in acute pancreatitis. Second, the C23 dosage utilized in this study (8 mg/kg body weight) was chosen based on the effective dose of C23 in animal models of sepsis and ischemia-reperfusion injury [33]. The optimal dose of C23 in acute pancreatitis remains to be determined. Third, C23 was administered at 2 h post-last L-arginine injection in this study. The therapeutic window of C23 in acute pancreatitis also warrants further investigation.

In summary, CIRP upregulation in AP promotes mitochondrial dysfunction and impairs mitochondrial autophagy through TLR4. This results in ROS accumulation, NLRP3-Caspase-1 pyroptosis signaling pathway activation, tissue damage, and systemic inflammation in AP. CIRP inhibition exerts protective effects on AP by improving mitochondrial function and suppressing pyroptosis in acinar cells. Based on our understanding, this is the first study to show that CIRP can directly induce pyroptosis in pancreatic acinar cells. Blocking CIRP could be a promising strategy idea for treating AP in the future.

## Materials and methods

### Experimental animals and L-arginine AP model

The Experimental Animal Center of Xi’an Jiaotong University provided male wild-type C57BL/6J mice. Subsequently, *CIRP* knockout (KO) mice, which were purchased from the Shanghai Model Organisms Center, were established by removing *CIRP* on a C57BL/6J background using the clustered regularly interspaced short palindrome repeat/caspase 9 technique. Animals were maintained at the Specific Pathogen-Free Center of Laboratory Animals, Xi’an Jiaotong University.

All mice (age, 8–10 weeks; bodyweight, 20–22 g) were allowed to fast for 12 h before the experiments. Two intraperitoneal doses of 4.0 g/kg L-arginine (A0013; Solarbio, Beijing, China) were utilized to induce L-Arginine-AP. In the pyroptosis inhibition group, a specific inhibitor for Caspase-1 (VX-765;200 mg/kg body weight, Selleck, S2228) was intraperitoneally injected into mice before the L-arginine injection and 1 h following the last L-arginine dose, respectively. In the ROS inhibition experiment, a specific scavenger of mitochondrial superoxide (MitoTEMPO, 20 mg/kg bodyweight; SML0737, Sigma, USA), 1 h before L-arginine injection. Furthermore, mice in the C23-treated group were administered with C23 (8 mg/kg bodyweight) at 2 h post-last L-arginine injection. An equivalent volume of physiological saline was administered intraperitoneally to the control animals.

Before the trials, all animals were kept for one week under standard parameters to achieve acclimation to the environment. Randomization was used to operate on all animals in an identical treatment. For this study, 18 mice (*n* = 6 per group) in total were utilized in the pyroptosis inhibition group. With six mice per group, the animals were categorized as indicated: (1)Control group (Sham); (2) Vehicle group(L-arg-AP); (3) VX765 group(L-arg-AP + VX765); The ROS inhibition experiment consisted of 12 mice (*n* = 6/group): (1) Vehicle group(L-arg-AP); (2) MitoTEMPO group (L-arg-AP+MitoTEMPO); The CIRP knockout group consisted of 24 mice (*n* = 6/group): (1) Control group (WT-Sham); (2) CIRP KO Control group (KO-Sham);(3) L-arg-AP group(WT-AP);(4) CIRP KO L-arg-AP group(KO-AP); The C23 administration group consisted of 16 mice (*n* = 4/sham group; *n* = 6/AP group): (1) Control group (Sham); (2) Vehicle group (WT-AP); (3) C23 group (WT-AP + C23);

We euthanized the animals 72 h after the initial injection of L-arginine. At 72 h following the initial injection of L-arginine, all mice were put under anesthesia by inhaling isoflurane. Furthermore, samples of blood and pancreatic tissue were collected. The Xi’an Jiaotong University Health Science Center Institutional Animal Care and Use Ethics Committee approved the research procedure.

### Cell culture

A Ham’s F-12K medium (PM150910, Procell, Wuhan, China) containing 20% fetal bovine serum (164210-500, Procell, Wuhan, China) was used to culture pancreatic AR42J cells (CL-0025, Procell, Wuhan, China) under humidified controlled conditions of 5% CO2 and 37 °C. Pancreatic AR42J cells used in the experiment were authenticated by STR profiling.AR42J cells (5 × 10^5^ cells/well) were subjected to recombinant CIRP (1.5 µg/mL) for 24 h or cerulein (10 nmol/L) + lipopolysaccharide (LPS; 10 µg/mL) for 24 h as indicated in the figure legends. In the MitoTEMPO-treated group, the ROS scavenger n-acetylcysteine (NAC; 5 nM) (BP907, Sigma, USA) was utilized for the pretreatment of pancreatic AR42J cells (5 × 10^5^ cells/well) for 2 h. Thereafter, pancreatic AR42J cells (5 × 10^5^ cells/well) in the C23-treated group were pretreated with C23 (450 ng/mL) (GRGFSRGGGDRGYGG synthesized from GenScript, Piscataway, NJ; dissolved in phosphate-buffered saline (PBS)) for 1 h. In the TAK242-treated group, pancreatic AR42J cells (5 × 10^5^ cells/well) were pretreated with TAK-242 (500 µM) for 24 h.

### Western blotting analysis

For the entire night at 4 °C, the membrane was treated with the primary antibodies (all 1:1000) listedbelow: anti-Caspase-1 (YT5743, Immunoway Biotechnology, USA); anti-Cleaved-Caspase-1 p20 (YC0022, Immunoway Biotechnology, USA); anti-ASC(DF6304, Affinity Biosciences, USA), anti-NLRP3 (BF8029, Affinity Biosciences, USA), anti-GSDMD N-terminal (YT7991, Immunoway Biotechnology, USA), anti-TFAM (ab176558, Abcam, USA), anti-PINK1 (#6946, CST, USA), anti-MFN2 (ab124773, Abcam, USA), LC3-II(#2775, CST, USA), anti-LAMP2A (#125068, Abcam, USA), and anti-β-ACTIN (#3700, CST, USA) antibodies. Next, the membrane was subjected to incubation for 45 min at 37 °C with horseradish peroxidase (HRP)-conjugated secondary antibodies (1:1000) (HRP-conjugated Affinipure Goat Anti-Mouse IgG, SA00001-1, Proteintech, USA and HRP-conjugated Affinipure Goat Anti-Rabbit IgG, SA00001-2, Proteintech, USA) at 37 °C for 45 min. Immunoreactive signals were developed utilizing a digital gel image analysis system (Bio-Rad, United States). Subsequently, the grayscale values of protein bands were determined using the ImageJ tool.

### Immunohistochemical (IHC) analysis

First, 4% formalin was utilized to fix pancreatic tissues before embedding them in paraffin. Next, IHC analysis was performed on the tissues with the anti-Caspase-1 (YT5743, Immunoway Biotechnology, USA) primary antibodies. Briefly, the paraffin blocks of pancreatic tissue were dewaxed, soaked, washed, and subjected to antigen recovery, followed by blocking them at room temperature for 20 min. The samples were subjected to incubation throughout the night with the primary antibodies at 4 °C, before being incubated for an hour with the corresponding secondary antibody at room temperature. Further, the samples were subjected to 3,3’-diaminobenzidine color development, hematoxylin staining, alcohol dehydration, cleaning, and gumming. The samples were imaged under a light microscope. For every image, three fields were selected at random. The immunoreactive signals were quantified using ImageJ software.

### Immunofluorescence staining

Pancreatic ROS was examined using dihydroethidium (DHE) staining (S0063, Beyotime, Shanghai, China), as directed by the manufacturer. Subsequently, the levels of ROS in pancreatic AR42J cells were examined using the ROS detection kit (S0033S Beyotime, Shanghai, China), per the directions stipulated by the manufacturer. For every image, three fields were selected at random, and images were obtained with a fluorescence microscope. We employed ImageJ to quantify the intensity of the fluorescent staining.

### Analysis of superoxide dismutase (SOD), glutathione (GSH)

The SOD and GSH levels in pancreatic tissues and AR42J cells were examined using the SOD assay kit (A001-3; Nanjing Jiancheng Bioengineering Institute, China) and the GSH assay kit (A006-2-1, Nanjing Jiancheng Bioengineering Institute, China), respectively, based on the guidelines stipulated by the manufacturers of the respective kits.

### Biochemical analysis

The serum amylase assay kit (C016-1, Nanjing Jiancheng Bioengineering Institute, Nanjing, China) was utilized to determine the serum amylase levels as guided by the manufacturer.

### Cell viability assay

The 3-(4,5-dimethylthiazol-2-yl)-2,5-diphenyl tetrazolium bromide (MTT) assay (G020-1-1, Nanjing Jiancheng Bioengineering Institute, Nanjing, China) was employed to determine cell viability in compliance with the recommendation of the manufacturer. Pancreatic AR42J cells (10^5^ cells/mL in 100 µL) were cultured in a 96-well plate and treated with recombinant CIRP (1.5 µg/mL) for 24 h. The following cell death inhibitors were used in this analysis: pyroptosis inhibitor, VX765 (5 µM; Selleck, S2228) (cells were pretreated with VX765 for 1 h); apoptosis inhibitor, Z-VAD-FMK (50 µM; Selleck, S7023) (cells were co-incubated); ferroptosis inhibitor, ferrostatin-1 (1 µM; Selleck, S7243) (cells were co-incubated). The optical density (OD) of samples at 570 nm was analyzed using an enzyme marker. Below is the formula for determining the survival rate: survival rate (%) = OD of the treatment group /OD of the control group(%).

### Histological evaluation of pancreatic injury

Histological analysis of the pancreas was performed using hematoxylin and eosin (HE) staining. Pancreatic tissue injury was evaluated utilizing Schmidt’s histological scoring system as previously described [53]. We employed a random selection process to select tissues from each group and three fields from each area to evaluate.

### Enzyme-linked immunosorbent assay (ELISA)

The tumor necrosis factor (TNF)-α, IL-6, lactate dehydrogenase (LDH), IL-1β, and CIRP levels were examined using the IL-1β (CSB-E08054m, CUSABIO, Wuhan, China), IL-6 (CSB-E08054m, CUSABIO, Wuhan, China), TNF-α (CSB-E04741m, CUSABIO, Wuhan, China), LDH (SEB864Mu, Cloud-Clone Corp USCN Life Science, Wuhan, China), and mouse cold-inducible RNA-binding protein ELISA kits (CSB-EL005440MO, CUSABIO, Wuhan, China), respectively.

### Transmission Electron Microscopy (TEM)

Staining with lead citrate and uranyl acetate was done on ultra-thin slices of pancreatic tissue samples measuring 70 nm. For TEM, 2.5% glutaraldehyde was utilized to fix approximately 1 mm^3^ pancreas tissues. Then the specimens were embedded into Epon by routine procedures. An electron microscope (HT7700, Hitachi, Japan) operated by a single technician was used to examine the ultrastructure of pancreatic mitochondria. Under the electron microscope, three domains were chosen at random for evaluation.

### Statistical analysis

The format of mean ± standard error of mean was utilized to represent the data. Means between groups were compared via a *t* test or one-way ANOVA test. The pathological score was analyzed via the Mann−Whitney U test (between 2 groups) and the Krukal-Wallis test(≥3 Groups). All statistical analyses were performed utilizing GraphPad Prism 8.0 software. We established a *p* < 0.05 as the significance criterion.

### Supplementary information

Supplementary material

## Data Availability

This manuscript contains all of the data that were generated or examined throughout the investigation. The corresponding author may provide the datasets upon reasonable request.
