# Supplementary material for: Blocking CIRP protects against acute pancreatitis by improving mitochondrial function and suppressing pyroptosis in acinar cells
Source: Cell Death Discov. 2024 Mar 27;10:156. doi: 10.1038/s41420-024-01923-6 (PMC10973482; doi:10.1038/s41420-024-01923-6)
Supplement: Supplementary file 1 — Supplementary material [file 41420_2024_1923_MOESM1_ESM.pdf]

**SUPPLEMENTARY MATERIALS**

# Blocking CIRP protects against acute pancreatitis by improving mitochondrial function and suppressing pyroptosis in acinar cells

Wuming Liu[1,2], Yifan Ren[1,3], Tao Wang[1,2], Mengzhou Wang[1,2], Yujia Xu[4], Jia Zhang[1,5], Jianbin Bi[1,6], Zheng Wu[2], Yuanyuan Zhang[7], Rongqian Wu[1]

[1] National Local Joint Engineering Research Center for Precision Surgery and Regenerative Medicine, Shaanxi Provincial Center for Regenerative Medicine and Surgical Engineering, The First Affiliated Hospital of Xi'an Jiaotong University, Xi'an, China

[2] Department of Hepatobiliary Surgery, The First Affiliated Hospital of Xi'an Jiaotong University, Xi'an, China

[3] Department of General Surgery, The Second Affiliated Hospital of Xi'an Jiaotong University, Xi'an, China

[4] Department of Pathology, The First Affiliated Hospital of Wenzhou Medical University, Wenzhou, China

[5] Department of Gastroenterology, The Second Affiliated Hospital of Xi'an Jiaotong University, Xi'an, China

[6] Department of Oncology, The Second Affiliated Hospital of Xi'an Jiaotong University, Xi'an, China

[7] Department of Pediatrics, The First Affiliated Hospital of Xi'an Jiaotong University, Xi'an, China

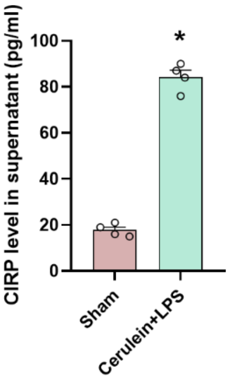


**Supplementary Figure 1. CIRP level in AR42J cells supernatant was upregulated *in vitro* AP model.**

Pancreatic AR42J cells ($5\times10^5$/well) were treated with Cerulein (10nmol/L) + LPS (10μg/mL) for 24h. CIRP level in AR42J cells supernatant. n=4/group. * p<0.05 versus sham group; Data are expressed as means ± SEM.

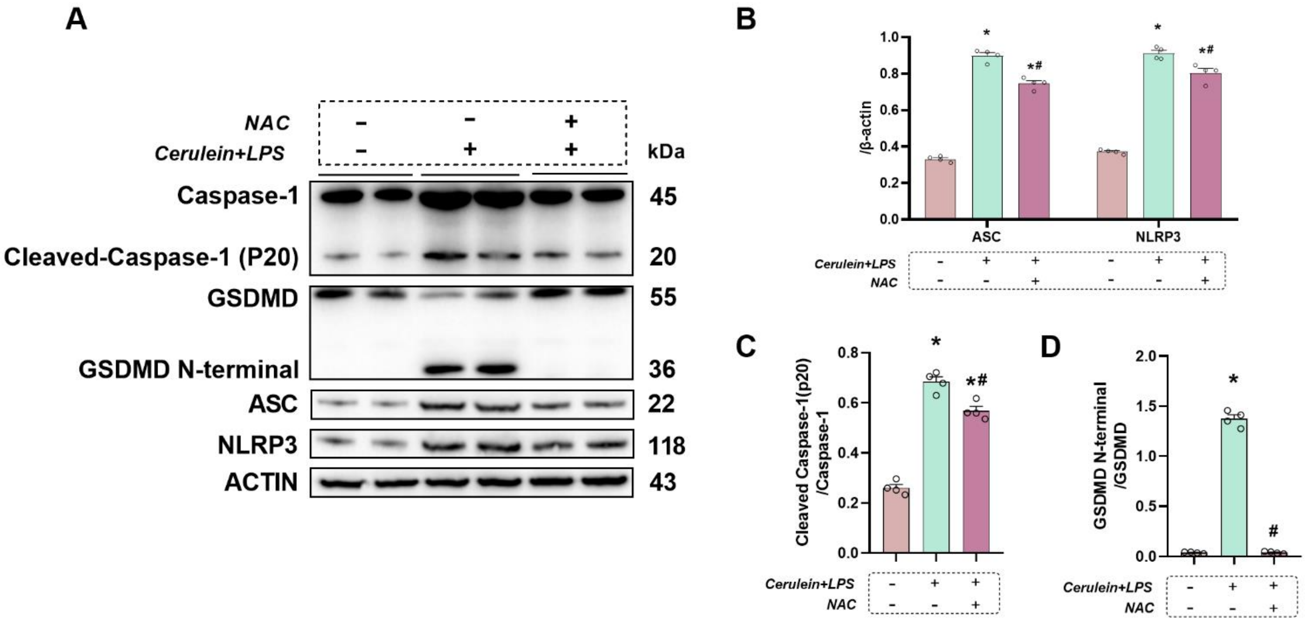


**Supplementary Figure 2. Inhibition of ROS reduced pancreatic pyroptosis *in vitro* AP model.**

Pancreatic AR42J cells ($5\times10^5$/well) were treated with Cerulein (10nmol/L) + LPS (10μg/mL) for 24h. The pancreatic AR42J cells were pretreated with ROS scavenger n-acetylcysteine (NAC,5nM) (BP907, Sigma, USA)

for 2h in the ROS inhibition group

(A-D) Western blot analysis of Caspase-1, Cleaved Caspase-1(P20), GSDMD, GSDMD N-terminal, ASC and NLRP3 in pancreatic AR42J cells treated with Cerulein + LPS for 24h with NAC or not. n=4/group. * $p<0.05$ versus sham group; # $p<0.05$ versus Cerulein+LPS group. Data are expressed as means ± SEM.

**Original Data: Unedited blot and gel images**

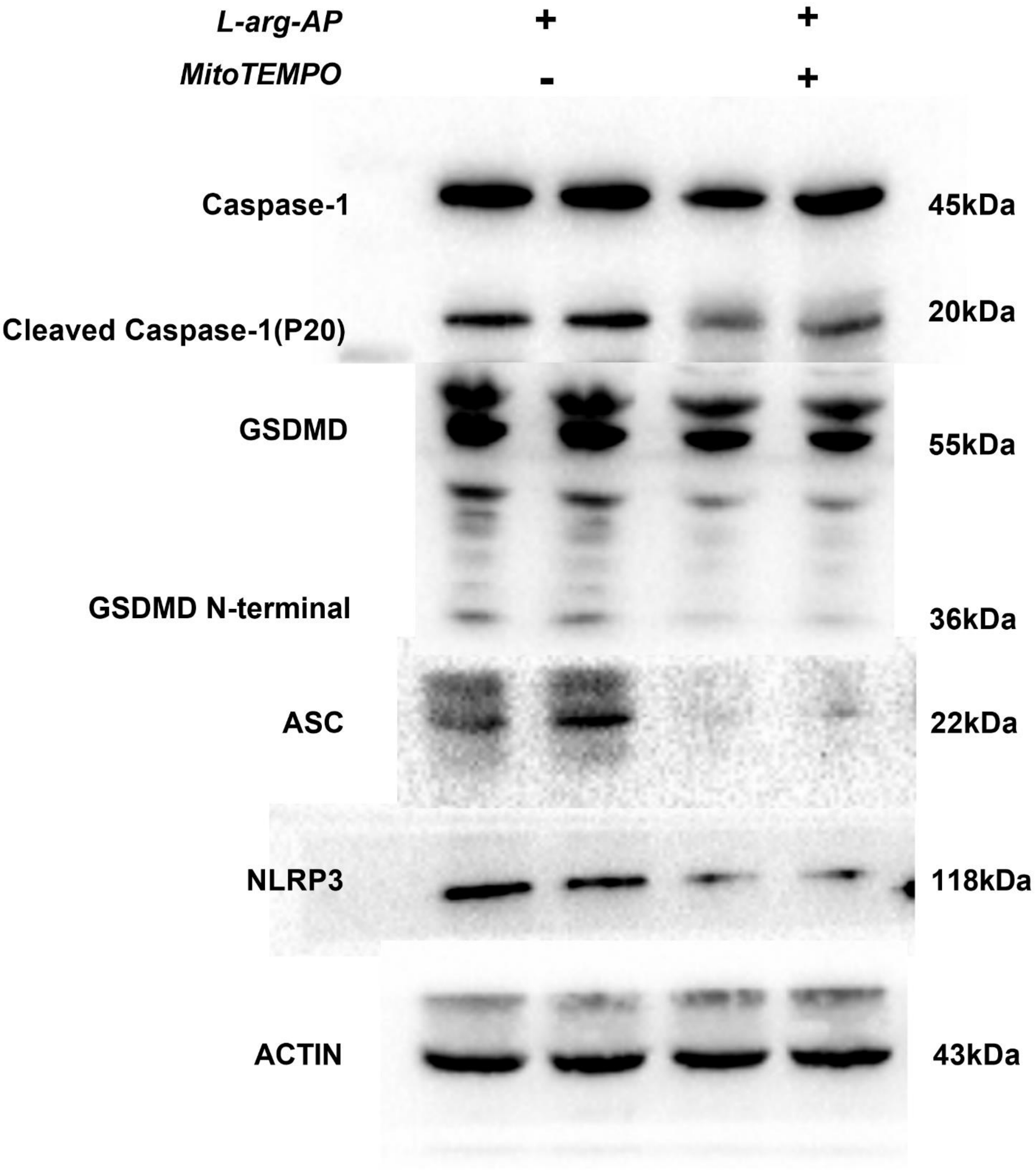


**Figure2F uncropped gel. Pancreatic tissue injury and pyroptosis were alleviated by ROS inhibition in L-arginine-induced acute pancreatitis.**

**Entire membranes of the representative Western blot of ACTIN, NLRP3, ASC, GSDMD N-terminal , GSDMD, Cleaved Caspase-1(P20) and Caspase-1, as shown in Figure 2F are provided. Of note, complete membranes were cut horizontally after protein transfer to allow for simultaneous incubation with different antibodies.**

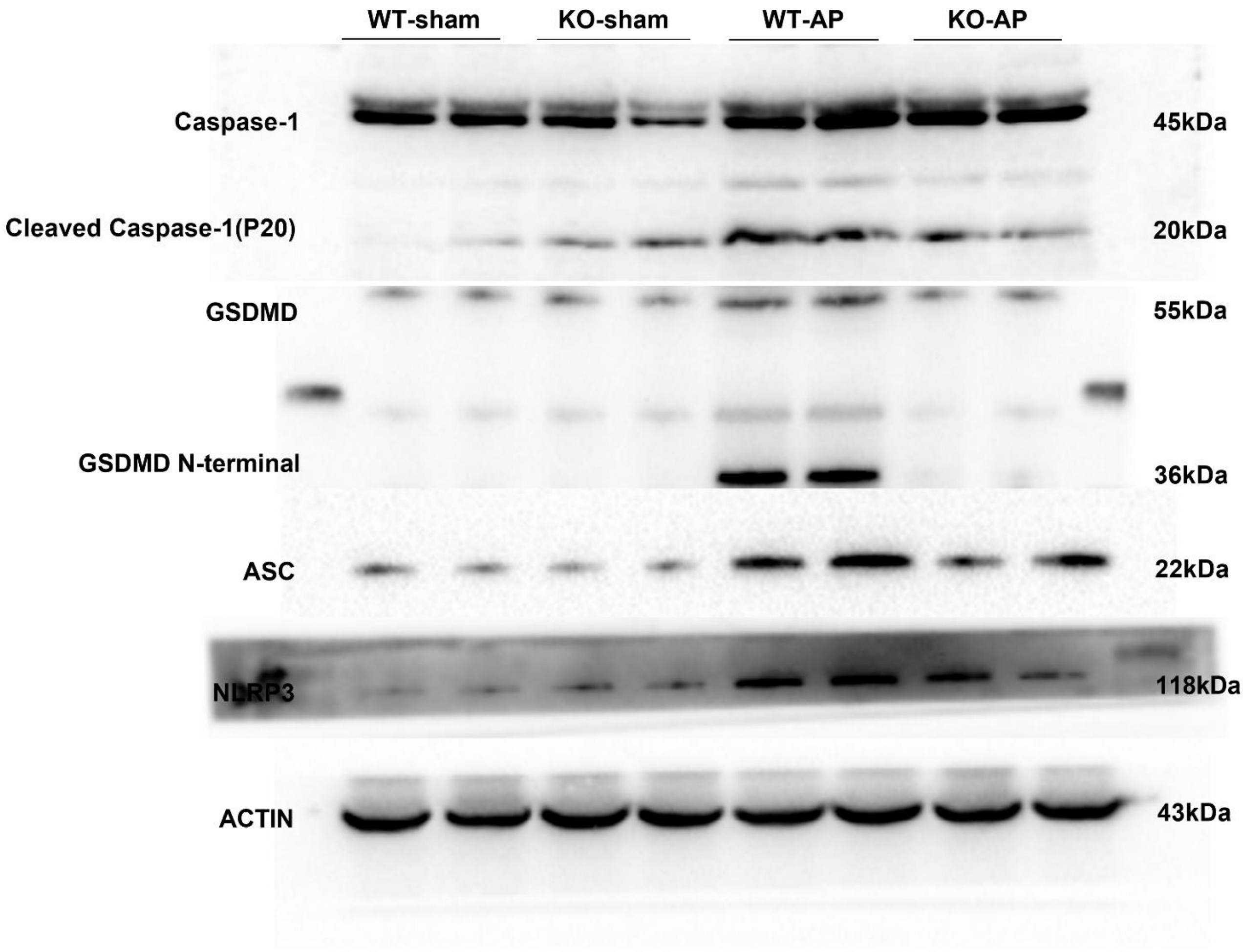


**Figure3D uncropped gel. CIRP KO attenuated pancreatic pyroptosis and tissue damage in the L-arginine-induced AP model.**

**Entire membranes of the representative Western blot of ACTIN, NLRP3, ASC, GSDMD N-terminal , GSDMD, Cleaved Caspase-1(P20) and Caspase-1, as shown in Figure 3D are provided. Of note, complete membranes were cut horizontally after protein transfer to allow for simultaneous incubation with different antibodies.**

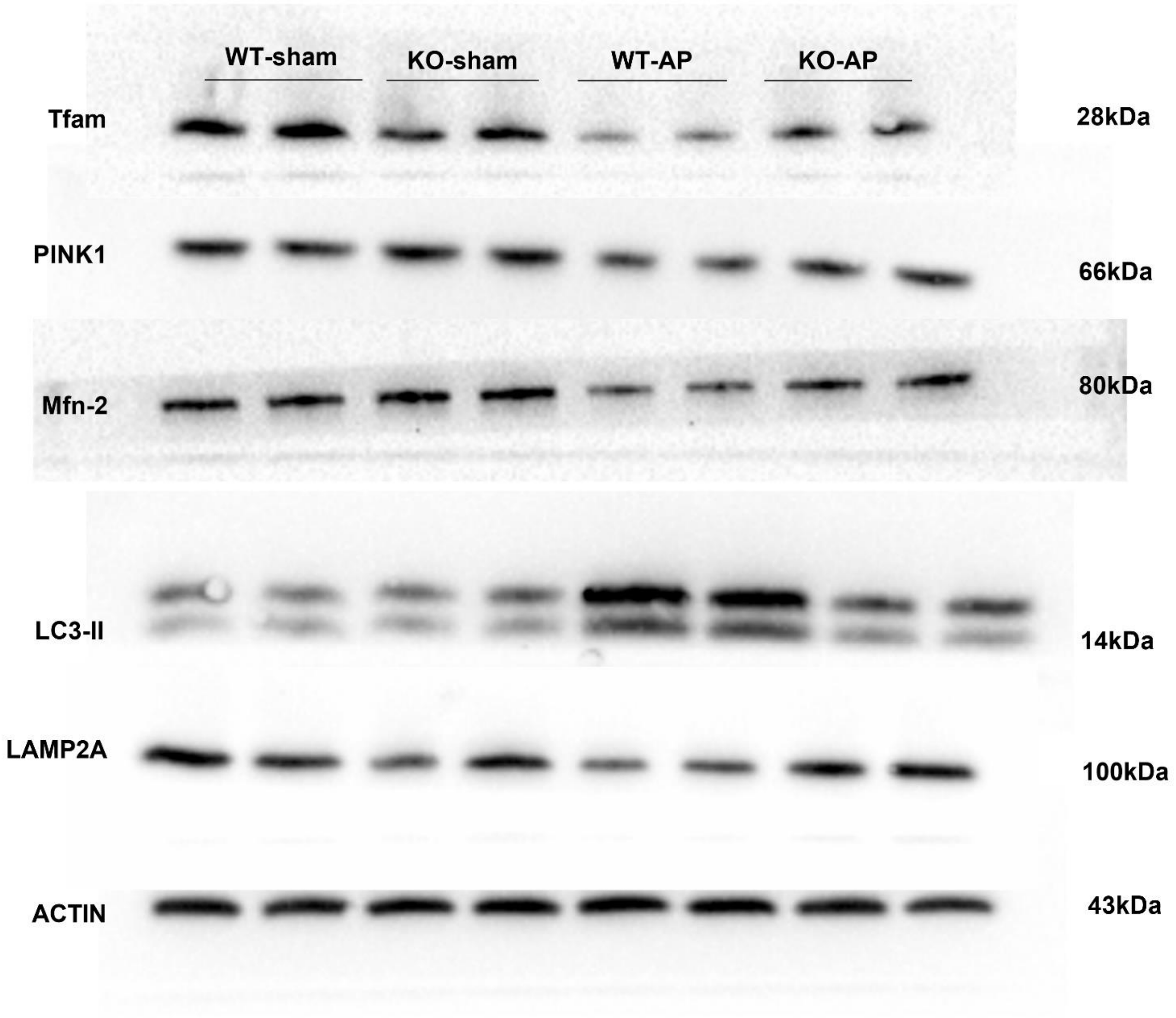


**Figure4D uncropped gel. CIRP knockout improved mitochondrial function and reduced ROS accumulation in the L-arginine-induced AP model.**

**Entire membranes of the representative Western blot of ACTIN, LAMP2A, LC3-II, Mfn-2, PINK1 and Tfam, as shown in Figure 4D are provided. Of note, complete membranes were cut horizontally after protein transfer to allow for simultaneous incubation with different antibodies.**

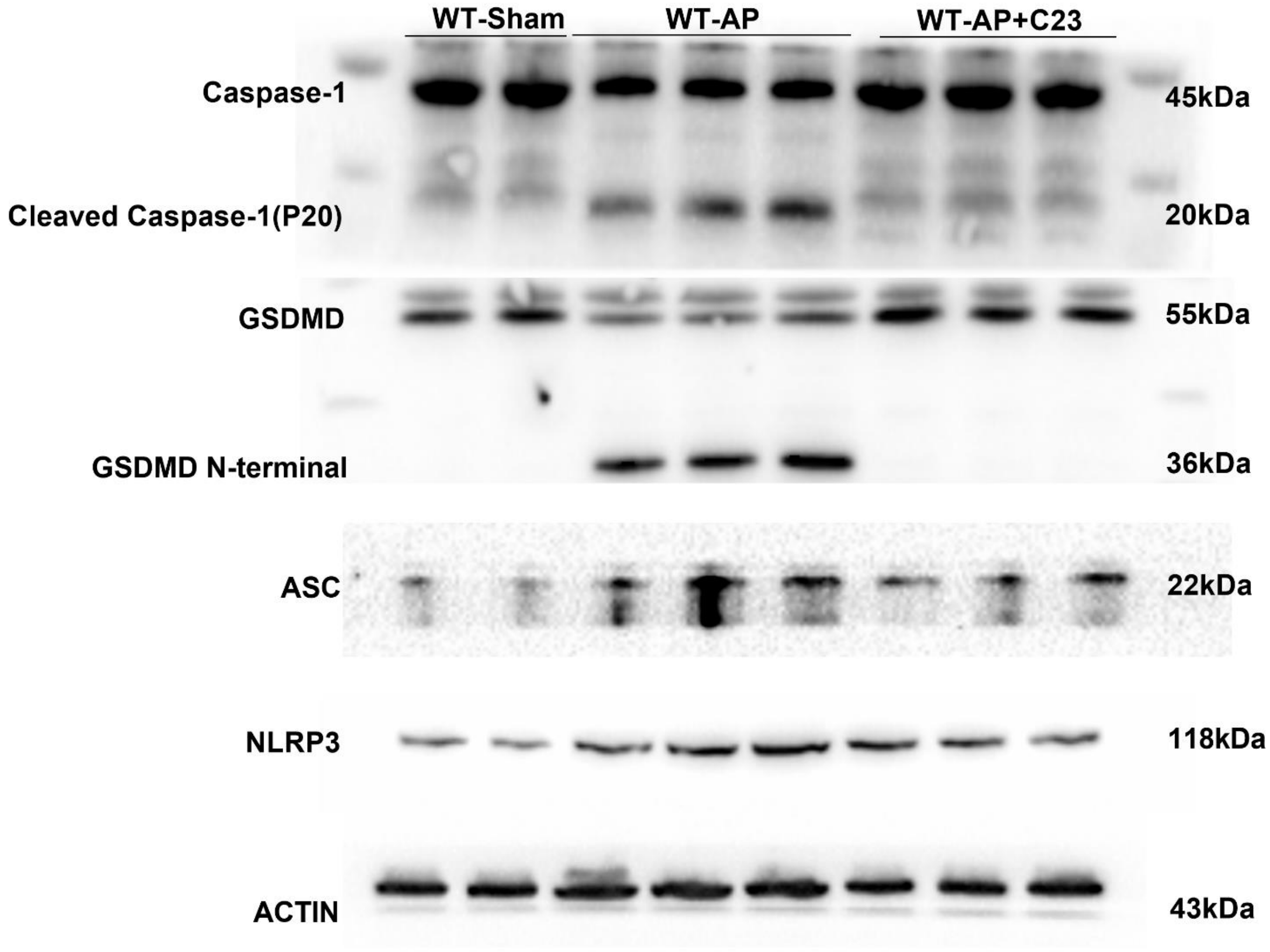


**Figure5C uncropped gel. C23 administration attenuated pancreatic pyroptosis and tissue damage in the L-arginine-induced AP model.**

**Entire membranes of the representative Western blot of ACTIN, NLRP3, ASC, GSDMD N-terminal , GSDMD, Cleaved Caspase-1(P20) and Caspase-1, as shown in Figure 5C are provided. Of note, complete membranes were cut horizontally after protein transfer to allow for simultaneous incubation with different antibodies.**

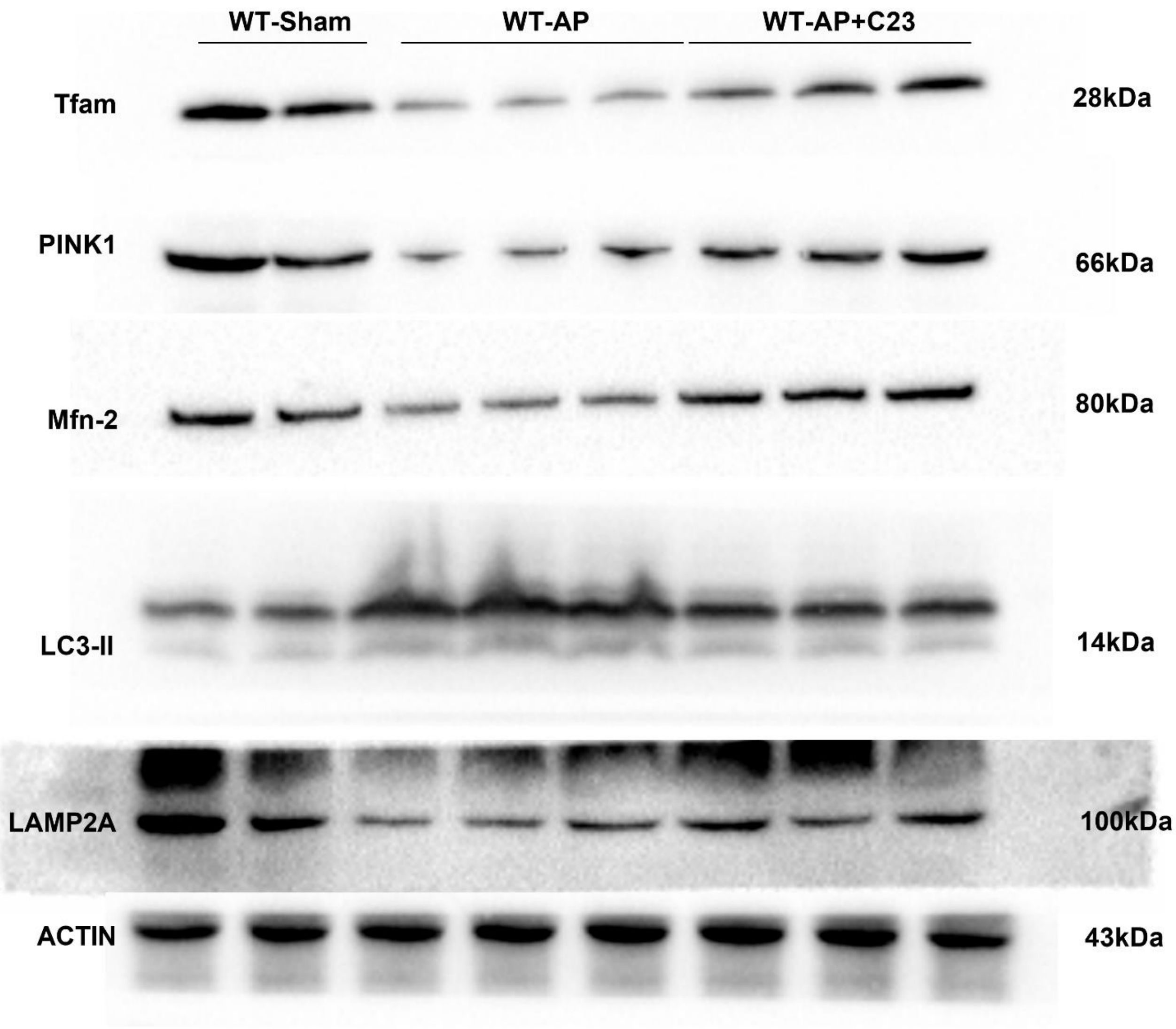


**Figure6D uncropped gel. C23 administration improved mitochondrial function and reduced ROS accumulation in the L-arginine-induced AP model.**

**Entire membranes of the representative Western blot of ACTIN, LAMP2A, LC3-II, Mfn-2, PINK1 and Tfam, as shown in Figure 6D are provided. Of note, complete membranes were cut horizontally after protein transfer to allow for simultaneous incubation with different antibodies.**

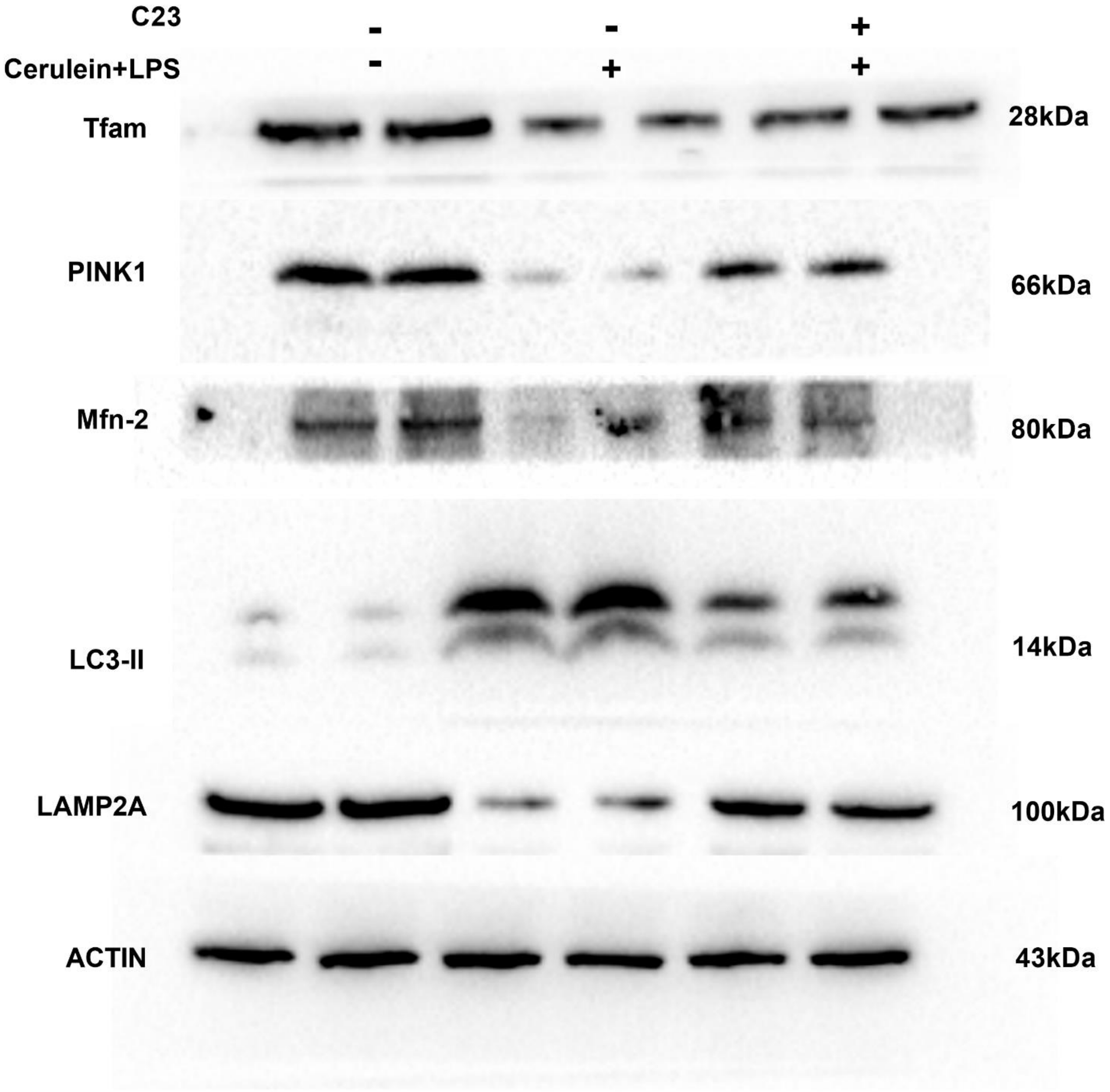


**Figure7A uncropped gel. C23 attenuated cerulein + LPS-induced mitochondrial dysfunction, autophagy impairment, ROS accumulation and pyroptosis in cultured pancreatic acinar cells.**

**Entire membranes of the representative Western blot of ACTIN, LAMP2A, LC3-II, Mfn-2, PINK1 and Tfam, as shown in Figure 7A are provided. Of note, complete membranes were cut horizontally after protein transfer to allow for simultaneous incubation with different antibodies.**

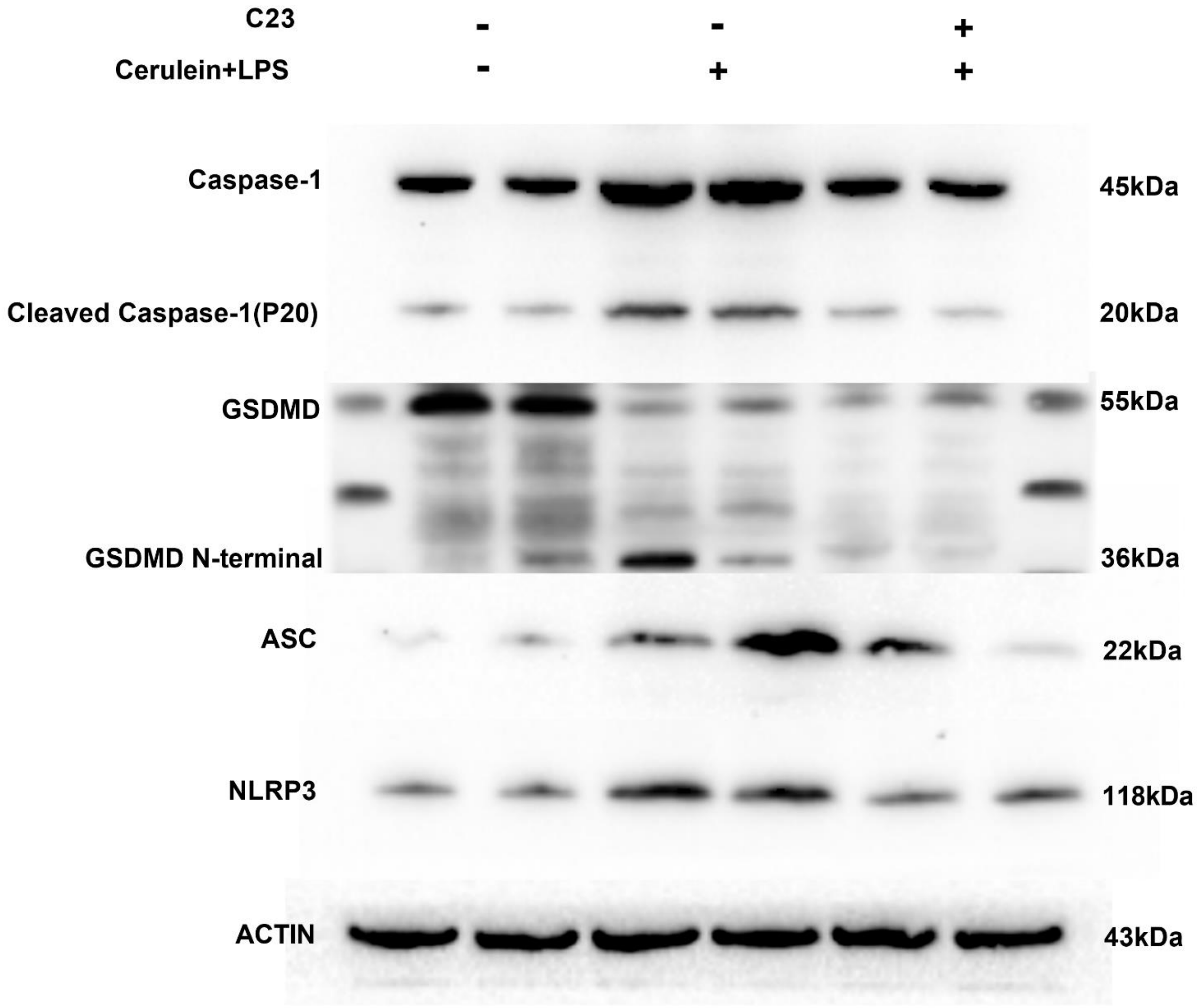


**Figure7F uncropped gel. C23 attenuated cerulein + LPS-induced mitochondrial dysfunction, autophagy impairment, ROS accumulation and pyroptosis in cultured pancreatic acinar cells.**

**Entire membranes of the representative Western blot of ACTIN, NLRP3, ASC, GSDMD N-terminal , GSDMD, Cleaved Caspase-1(P20) and Caspase-1, as shown in Figure 7F are provided. Of note, complete membranes were cut horizontally after protein transfer to allow for simultaneous incubation with different antibodies.**

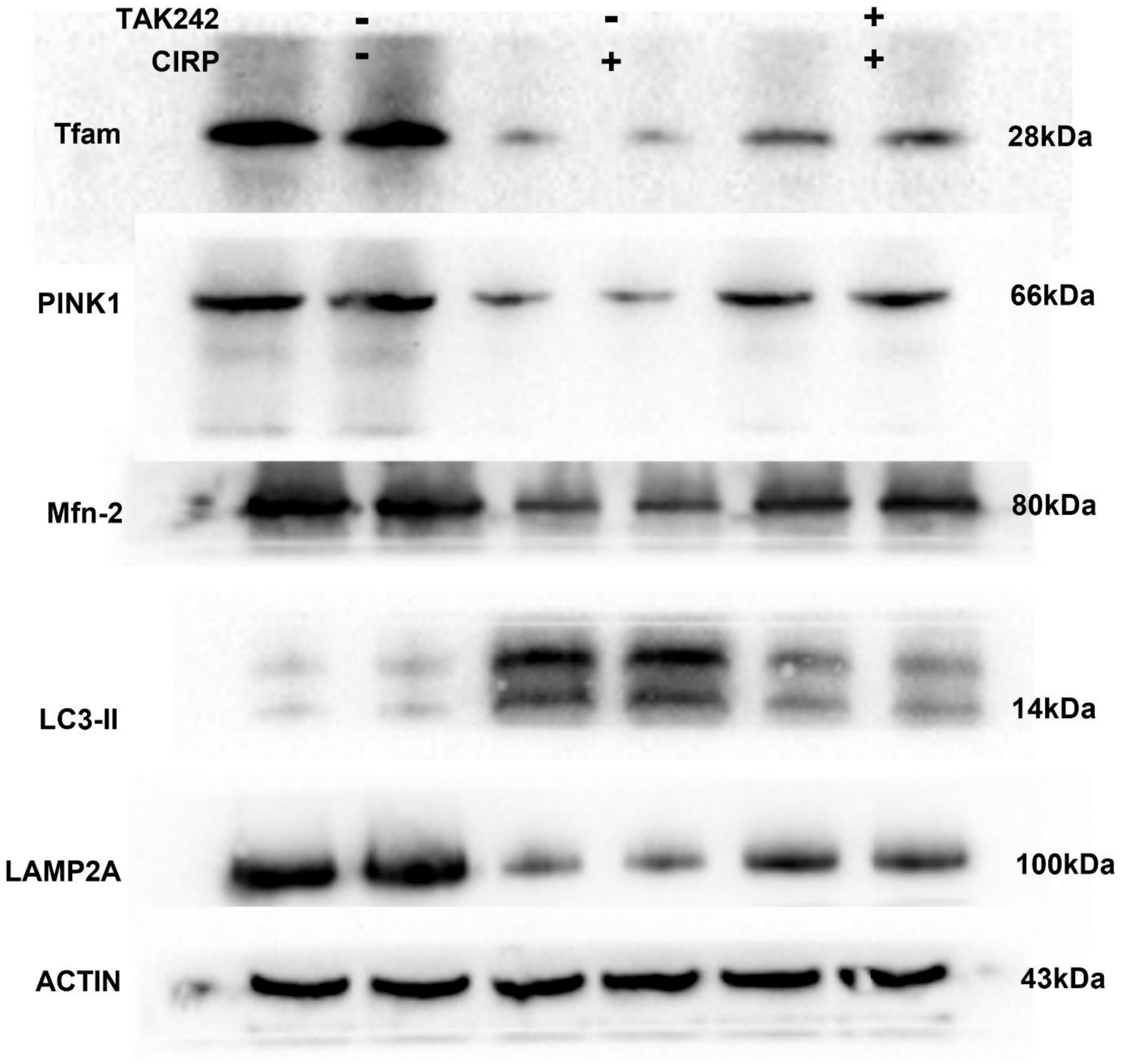


**Figure8A uncropped gel. CIRP directly induced mitochondrial dysfunction, autophagy impairment, ROS accumulation and pyroptosis in cultured pancreatic acinar cells.**

**Entire membranes of the representative Western blot of ACTIN, LAMP2A, LC3-II, Mfn-2, PINK1 and Tfam, as shown in Figure 8A are provided. Of note, complete membranes were cut horizontally after protein transfer to allow for simultaneous incubation with different antibodies.**

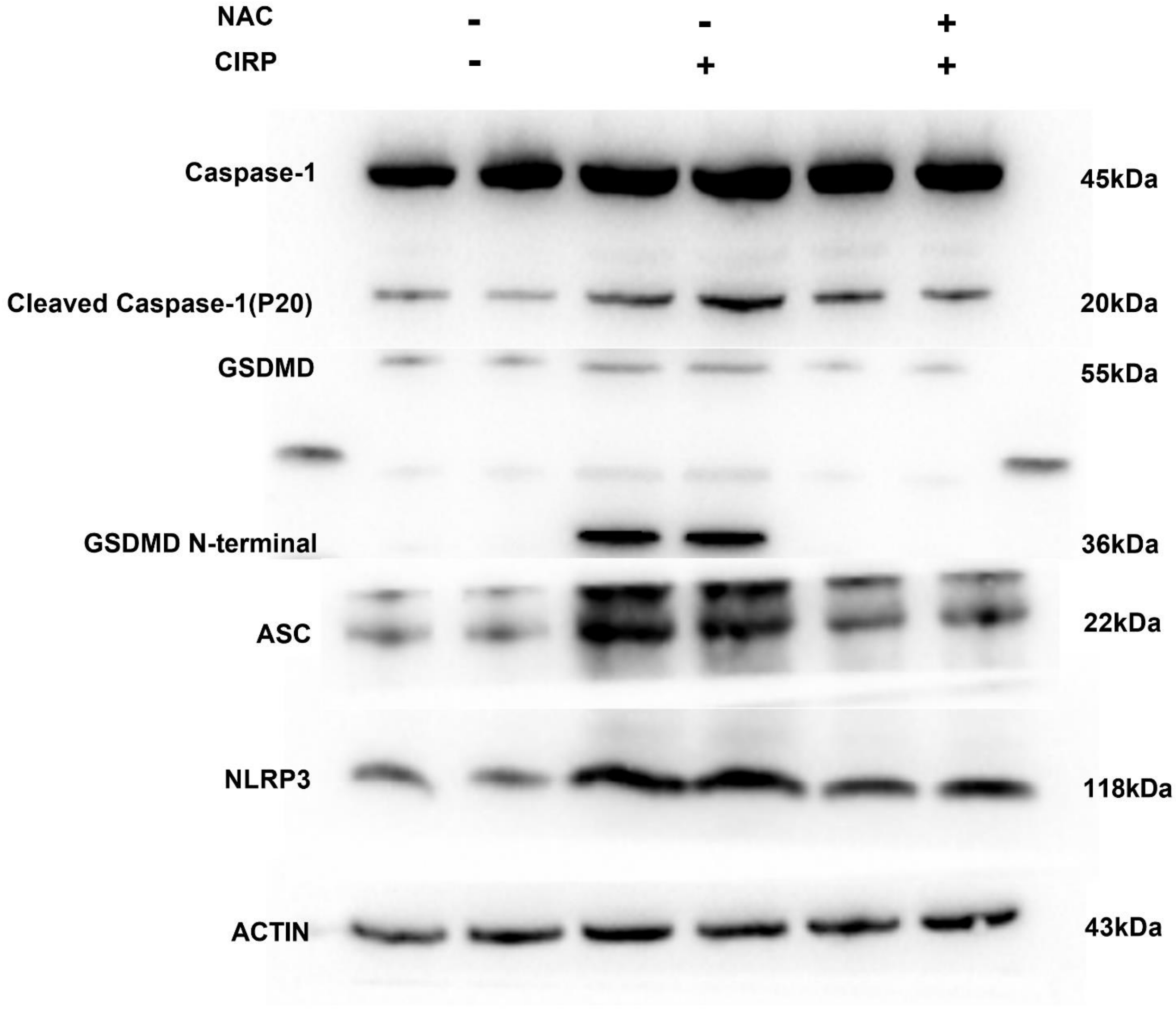


**Figure8F uncropped gel. CIRP directly induced mitochondrial dysfunction, autophagy impairment, ROS accumulation and pyroptosis in cultured pancreatic acinar cells.**

**Entire membranes of the representative Western blot of ACTIN, NLRP3, ASC, GSDMD N-terminal , GSDMD, Cleaved Caspase-1(P20) and Caspase-1, as shown in Figure 8F are provided. Of note, complete membranes were cut horizontally after protein transfer to allow for simultaneous incubation with different antibodies.**

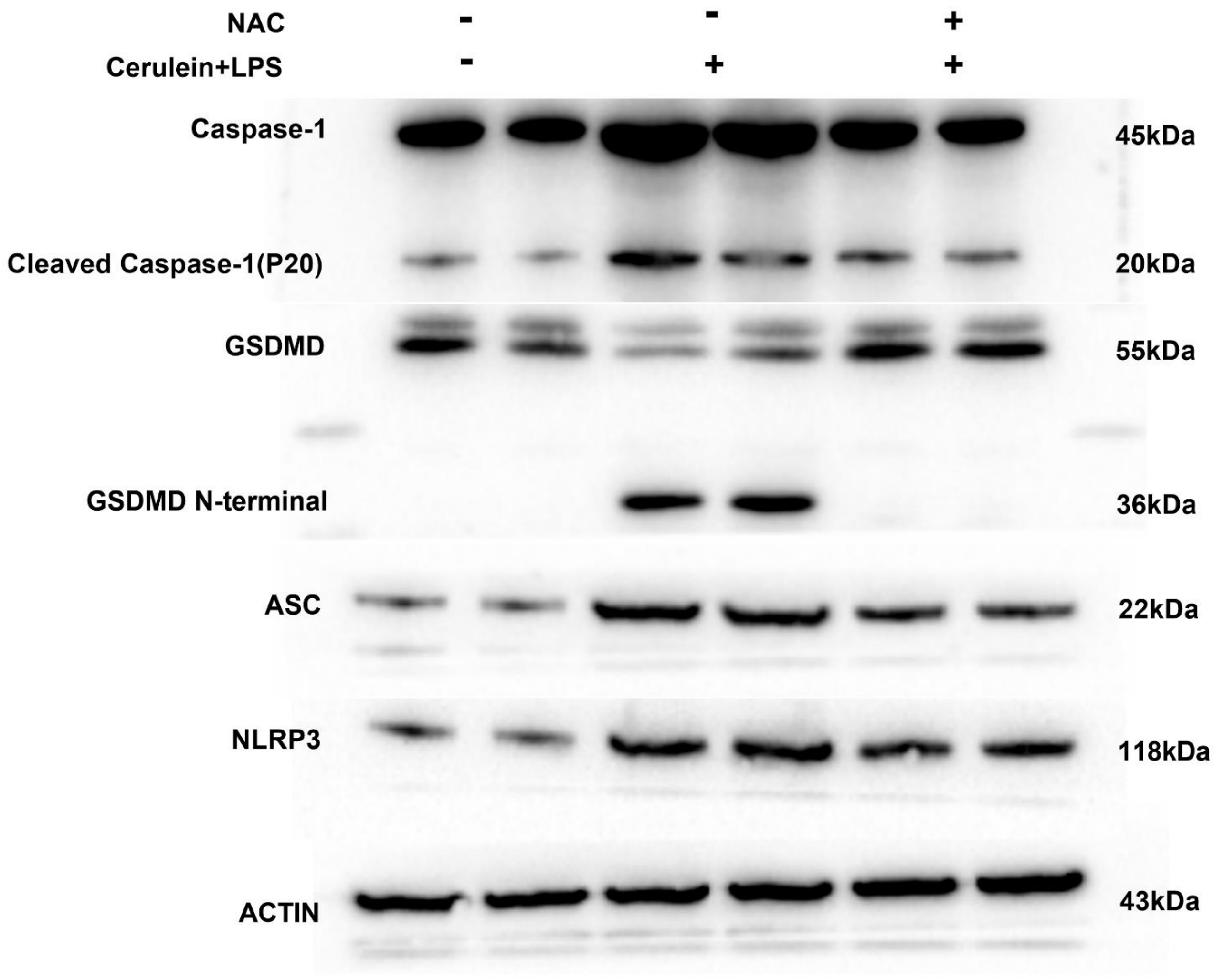


**Supplementary Figure1B uncropped gel. Inhibition of ROS reduced pancreatic pyroptosis *in vitro* AP model.**

**Entire membranes of the representative Western blot of ACTIN, NLRP3, ASC, GSDMD N-terminal, GSDMD, Cleaved Caspase-1(P20) and Caspase-1, as shown in Supplementary Figure1B are provided. Of note, complete membranes were cut horizontally after protein transfer to allow for simultaneous incubation with different antibodies.**

## Author Contribution Statement

Wuming Liu compiled the data, analyzed them, and drafted the article.

Yifan Ren, Tao Wang, Mengzhou Wang, and Yujia Xu participated in data acquirement.

Zheng Wu analyzed the results.

Yuanyuan Zhang interpreted the data and revised the manuscript.

Rongqian Wu designed and supervised the study and revised the manuscript. Yuanyuan Zhang(Email: yuanyuanzhang@xjtu.edu.cn) and Rongqian Wu(Email:rwu001@mail.xjtu.edu.cn.) are co-corresponding authors.

After reading the final manuscript, each author provided their approval.
